# Phylogeny and Metabolic Potential of the Candidate Phylum SAR324

**DOI:** 10.3390/biology11040599

**Published:** 2022-04-14

**Authors:** Lukas Malfertheiner, Clara Martínez-Pérez, Zihao Zhao, Gerhard J. Herndl, Federico Baltar

**Affiliations:** 1 Department of Functional and Evolutionary Ecology, University of Vienna, 1090 Vienna, Austria; lukas.malfertheiner@uzh.ch (L.M.); martinez@ifu.baug.ethz.ch (C.M.-P.); zihao.zhao@univie.ac.at (Z.Z.); gerhard.herndl@univie.ac.at (G.J.H.); 2 Institute for Environmental Engineering, Department of Civil, Environmental and Geomatic Engineering, Eidgenössische Technische Hochschule (ETH) Zürich, 8093 Zurich, Switzerland; 3 NIOZ, Department of Marine Microbiology and Biogeochemistry, Royal Netherlands Institute for Sea Research, Utrecht University, 1790 AB Den Burg, The Netherlands

**Keywords:** microbial ecology, metagenomics, comparative genomics, phylogeny, metabolism, nutrient cycling, extreme environments

## Abstract

**Simple Summary:**

SAR324, newly proposed as its own candidate phylum, is a diverse and globally abundant bacterial group living in a wide range of environments, from deep-sea hydrothermal vents and brine pools to the epipelagic regions of the global oceans and terrestrial aquifers. The different SAR324 clades harbor a diverse array of genes and pathways well adapted to their respective environments. This metabolic flexibility explains the ubiquitous presence and the importance of SAR324 in global biogeochemical cycles.

**Abstract:**

The bacterial SAR324 cluster is ubiquitous and abundant in the ocean, especially around hydrothermal vents and in the deep sea, where it can account for up to 30% of the whole bacterial community. According to a new taxonomy generated using multiple universal protein-coding genes (instead of the previously used 16S rRNA single gene marker), the former *Deltaproteobacteria* cluster SAR324 has been classified since 2018 as its own phylum. Yet, very little is known about its phylogeny and metabolic potential. We downloaded all publicly available SAR324 genomes (65) from all natural environments and reconstructed 18 new genomes using publicly available oceanic metagenomic data and unpublished data from the waters underneath the Ross Ice Shelf. We calculated a global SAR324 phylogenetic tree and identified six clusters (namely 1A, 1B, 2A, 2B, 2C and 2D) within this clade. Genome annotation and metatranscriptome read mapping showed that SAR324 clades possess a flexible array of genes suited for survival in various environments. Clades 2A and 2C are mostly present in the surface mesopelagic layers of global oceans, while clade 2D dominates in deeper regions. Our results show that SAR324 has a very versatile and broad metabolic potential, including many heterotrophic, but also autotrophic pathways. While one surface water associated clade (2A) seems to use proteorhodopsin to gain energy from solar radiation, some deep-sea genomes from clade 2D contain the complete Calvin–Benson–Bassham cycle gene repertoire to fix carbon. This, in addition to a variety of other genes and pathways for both oxic (e.g., dimethylsulfoniopropionate degradation) and anoxic (e.g., dissimilatory sulfate reduction, anaerobic benzoate degradation) conditions, can help explain the ubiquitous presence of SAR324 in aquatic habitats.

## 1. Introduction

Microbes are key players in the biogeochemical nutrient cycling of the ocean, the biggest habitat on Earth [1,2]. The most abundant, culturable bacterial clades in surface waters, such as the marine *Roseobacter* clade, SAR11 and the genus *Prochlorococcus*, have been extensively studied in the last few decades, and their interactions and roles in the microbial loop are well established [3,4,5,6,7,8]. However, most bacteria in the ocean remain uncultured, and until recent molecular advances (e.g., next-generation sequencing techniques), it was impossible to obtain profound knowledge about uncultivable bacteria [9]. The first application of shotgun metagenomic sequencing to marine samples more than a decade ago revealed an incredible diversity of microbes in surface but also deep waters [10]. The deep ocean is, in terms of volume, the largest habitat in the biosphere. Yet, although recent studies suggest a major impact of these deep-sea bacteria and archaea on the global biogeochemical cycling [2,11], the difficulties and costs associated with deep-ocean sampling, together with the ability to obtain pure cultures of most of its resident microbes, preclude a deep understanding of the functional diversity of deep-ocean microbes. Thanks to recent novel bioinformatic tools to recover genomes from metagenomic samples, it is now possible to investigate the functional potential of key players in this intriguing environment [12,13]. Especially in the light of global change, it is crucial to investigate these microbial communities and their metabolic potential [14,15,16].

One of the bacterial clades that is ubiquitous in the marine environment and especially abundant in the deep layers is SAR324, previously known as marine group B [17]. The first 16S rRNA sequences of SAR324 were identified in 1997 from mesopelagic Sargasso Sea samples [18]. Especially in the bathypelagic region, SAR324 is consistently among the five most abundant operational taxonomic units (OTUs), contributing up to >30% of the bacterial community in oceanic basins globally [19]. While previously classified as a deep-branching Deltaproteobacteria clade, the use of 120 bacterial marker genes for the generation of a revised phylogenetic tree recently suggested that SAR324 is a separate phylum [20]. Despite their global presence, relatively few studies have investigated their phylogeny and metabolic potential. Among the limited known features of deep-ocean SAR324 revealed by single-cell amplified genomes (SAG) include the presence of ribulose-1,5-bisphosphate carboxylase-oxygenase (RuBisCO)—a key enzyme mediating the Calvin–Benson–Bassham (CBB) cycle and evidence of a potential particle-attached lifestyle [21]. Two further studies investigating metagenomic-assembled genomes from hydrothermal vents and surrounding waters confirmed the presence of RuBisCO and also proposed an alternative complex III in the electron transport chain [22,23]. Additionally, SAR324 was recently identified as a potential major contributor to both carbon fixation and incorporation in the North Atlantic Deep Water [24]. A recent pangenomic study shed light on SAR324 in the different depth layers of the North Pacific Subtropical Gyre, revealing a depth-dependent shift in their metabolic potential [25]. It appears that SAR324 is split into different eco-groups, showing characteristic features depending on the depth of their occurrence, such as the presence of proteorhodopsin in surface-associated SAR324 and the CBB cycle in the deep ocean [25]. Still, very little is known about the functional diversity of SAR324 in other marine regions and how they compare to non-marine environments, precluding a deeper understanding of the role of this phylum from a global perspective.

Hence, we collected all available genomes from databases classified as SAR324 and additionally produced and extracted new metagenome-assembled genomes (MAGs, for simplicity, also referred to as genomes) from natural samples to obtain an overview of the abundance, phylogeny and metabolic potential of this novel phylum on a global scale. To achieve this, we used a pangenomic approach to understand the similarities and differences of different SAR324 clusters and determine their ecological impact and the reasons for their success in populating diverse habitats. We hypothesized that the environment played a key role in the phylogenetic and functional diversity of SAR324 subclusters. We also hypothesized to find a wide metabolic versatility of this clade, potentially explaining its widespread distribution and relevance.

## 2. Material and Methods

### 2.1. Determining the Relative 16S rRNA Gene Sequence Abundance of SAR324 in the Oceanic Water Column

The relative sequence abundance of SAR324 in epipelagic and mesopelagic ocean waters was obtained from 16S rDNA miTAG Illumina sequence data present as a fasta file for 138 TARA Ocean samples [26]; miTAGs were also extracted from bathypelagic samples from the Malaspina Circumnavigation expedition [27] metagenomic surveys in the Arctic and the Southern Ocean [28], as well as metagenomic datasets from polar regions obtained from the TARA Ocean Expedition [29] and the waters underneath the Ross Ice Shelf [30] using a previously described protocol [31]. Extracted 16S rDNA reads were mapped to the SILVA non-redundant SSU Ref database (v.138) [32] and assigned to an approximate taxonomic affiliation (nearest taxonomic unit, NTU) using PhyloFlash v.3.0 [33]. Counts per NTU (at phylum-level resolution) of extracted miTAGs were summarized with the ggplot2 package [34] in R. Only bacterial and archaeal species with >4 reads per sample were included in the analyses. Samples were divided into three groups, according to sampling depth: epipelagic (depth < 200 m, *n* = 169), mesopelagic (depth ~200–1000 m, *n* = 69) and bathypelagic (depth 1000–4000 m, *n* = 54).

### 2.2. De Novo Assembly and Binning of SAR324 Genomes from under the Ross Ice Shelf (RIS)

Reads were quality checked by using FastQC [35]. Paired-end reads were error corrected with SPAdes 3.12.0 [36], merged with BBmerge v.36.32 and normalized to a kmer depth of 42 with BBnorm v.36.32 from the BBtools program suite (https://sourceforge.net/projects/bbmap/, accessed on 13 April 2022). MEGAHIT v.1.1.1 [37] was used to co-assemble the metagenomes with merged and unmerged reads (--k-min 21 --k-max 121 --k-step 10). Only contigs with more than 1000 bp length were used for binning and downstream analysis. The raw reads were mapped to the contigs with BBmap 37.61 with an absolute cut-off at 95% (minid = 97 idfilter = 95) to estimate the coverage of the contigs in the different samples. Contigs from the assemblies were separately binned using the three binning programs: MetaWatt 3.5.3 [38], MetaBAT 2.12.1 [39] and MaxBin 2.2.4 [40] with standard parameters. DASTool v 1.1.0 (--score_threshold 0.2) was used to refine and combine the resulting bins [41]. CheckM 1.0.7 [42] was used to check completeness and contamination values. Furthermore, bins were manually checked and corrected for contamination using anvio-interactive v 5.2 [43]. Unbinned contigs from this first approach were classified taxonomically with diamond [44].

### 2.3. Obtaining Existing SAR324 Genomes and Re-Assembly and Binning of SAR324 Genomes from Metagenomic Collections

All publicly available genomes classified as SAR324 were downloaded from the genome taxonomy DataBase (gtdb) rRelease 03-RS86. The obtained bins and unbinned contigs assigned to SAR324 from public databases and the Ross Ice Shelf (RIS) were used as a reference database for read recruitment with BBmap 37.61 (using standard parameters) in the Malaspina and the RIS datasets as well as a deep-sea assembly from the North Pacific Ocean [45]. Mapped reads were assembled using SPAdes 3.12.0 with the --meta option and --only-assembler enabled and k-mer sizes of 21, 31, 41, 51, 61, 71, 81, 91, 101, 111 and 121. Mapped reads were also assembled with MEGAHIT 1.1.2 as described for the first assembly in the previous paragraph to select the best bins from either assembly strategy. Only contigs with a length >1000 bp were used for binning and downstream analysis. The raw reads were mapped to the contigs with BBmap 37.61 with an absolute cut-off at 95% (minid = 97 idfilter = 95) to estimate the coverage of the contigs in the different samples. Contigs from both assemblies were separately binned using the three binning programs: MetaWatt 3.5.3 [38], MetaBAT 2.12.1 [39] and MaxBin 2.2.4 [40] with standard parameters. DASTool v 1.1.0 (--score_threshold 0.2) was used to refine and combine the resulting bins [41]. CheckM 1.0.7 [42] was used to check completeness and contamination values. Furthermore, bins were manually checked and corrected for contamination using anvio-interactive v 5.2 [43].

### 2.4. De-Replication and Genome Characteristics

The total number of 72 bins (i.e., 3 out of public Malaspina samples, 2 from under the Ross Ice Shelf, 1 from a deep-sea sample in the North Pacific Ocean, 2 from a brine pool in the Red Sea, 64 downloaded from gtdb) were de-replicated using dRep 1.4.3 [46]. We used maximum contamination of 5%, minimum completeness of 64% and the additional parameters -nc 0.25 -sa 0.985 to eliminate identical genomes and reduce redundancy. Since the RIS is a novel and understudied environment, we decided to additionally include RIS_MetaBAT_11f, although it has not been originally selected as the best genome in its cluster by dRep. The resulting 25 genomes were used for downstream analysis. Graphical visualization of the de-replication can be found in Supplementary Figure 1. The characteristics of the remaining genomes were accessed using a combination of the stats.sh command in BBmap 37.61 and CheckM 1.0.7. To determine statistical differences between the GC-content of different clades, we used a two-tailed Student’s *t*-test considering unequal variance in the base version of R.

### 2.5. Phylogeny Clustering

All resulting 25 genomes were screened for 16S rRNA sequences with the Hidden Markow Model (HMM) based program metaRNA [47]. All the detected sequences were extracted, and 16S rRNA sequences with a length >400 bp were classified and aligned using the SILVA 123 release reference library [32]. Additionally, closely related SAR324 sequences from the SILVA database were downloaded, and the resulting alignment was used to calculate a maximum likelihood phylogenetic tree with 100 bootstrap support with IQtree [48]. The phylogeny of the genomes was inferred using part of the de_novo_wf workflow in GTDB-Tk v0.2.1 [20], based on a set of 120 bacterial single-copy marker genes and IQtree [48]. Three genomes belonging to different families of the phylum *Myxococcota* were downloaded to serve as an outgroup (GCF_900111765.1, GCF_000418325.1, GCF_006517175.1). The marker genes were extracted and aligned following the identify and align steps of gtdbtk, and the resulting multiple alignment was used to construct a maximum likelihood tree in IQtree with the -B 1000 parameter to assess branch support with ultrafast bootstrap approximation [49]. The resulting tree was rooted with *Myxococcota* as an outgroup. The candidate phylum SAR324 was split into different clades, taking into account the phylogenetic clustering as well as environmental information. Most of the assigned clades overlapped with the suggested taxonomy of the GTDB and were renamed for clarity. Here, o__XYD2-FULL-50–16 corresponds to Group 1 and o__SAR324 corresponds to Group 2. The single exception was GCA_002753255 (originally belonging to o__SAR324), which was placed into Group 1 to have a clear environmental separation (marine/non-marine) of Group 1 and 2 (Figure 2). The tree was manually illustrated using the ggtree package [50] in R and annotated with metadata about the origin and alternative names of the resulting clades.

### 2.6. Annotation and Database Generation

For annotation, open reading frames were identified using Prodigal [51]. All the gene-coding amino acid sequences were annotated with the myRAST pipeline [52]. Annotations were verified with Interproscan v. 5.31–70.0, appl = Pfam,Phobius,TIGRFAM,Hamap,ProSitePatterns,ProSiteProfiles [53]. Hydrogenases were additionally uploaded to the HydDB to determine their exact function and group [54]. Anvi’o v 5.2 [43] was used to create a database for each individual genome and import the annotations (anvi-import-functions). The annotated single-genome databases were then combined using the Anvi’o pangenome workflow [55]. The pangenomic analysis (anvi-pan-genome) used the default parameters for minbit69 (-minbit 0.5) and MCL70 (-mcl-inflation 2) for the generation of protein clusters. An Anvi’o script (anvi-get-enriched-functions-per-pan-group) was performed to determine putatively enriched functions or proteins in the different SAR324 clades. A custom-made python script was used to obtain a presence/absence matrix for key proteins and pathways of interest based on the myRast-annotations from the final pangenomic database. Genes that were significantly different between the SAR324 clusters based on the Anvi’o script and other genes of interest were examined in detail and summarized in a heatmap created in R with the heatmap.2 package.

### 2.7. Mapping to TARA Ocean Metatranscriptome

The publicly available TARA Ocean metatranscriptomes from 495 different sample sites were mapped to the 25 generated genomes. The reads were downloaded according to the script from Salazar et al. [29]; (https://github.com/SushiLab/omrgc_v2_scripts/blob/master/analysis/Download_data.sh, accessed on 13 April 2022). Burrows–Wheeler Aligner [56] was used to align reads to corresponding MAGs (bwa mem). The SAM files were further converted into a relative abundance table and coverage table using bbmap (pileup.sh) [57]. The resulting counts of mapped reads were filtered to only include genes with a mapped coverage of >95%. Thereafter, the percentage of mapped reads belonging to previously established SAR324 clades in all samples within the respective depth layers was calculated using a combination of a custom-made R and python script and visualized using the R library ggplot 2.

## 3. Results and Discussion

### 3.1. Distribution and Relative Sequence Abundance of SAR324 in the Ocean

**Figure 1:**
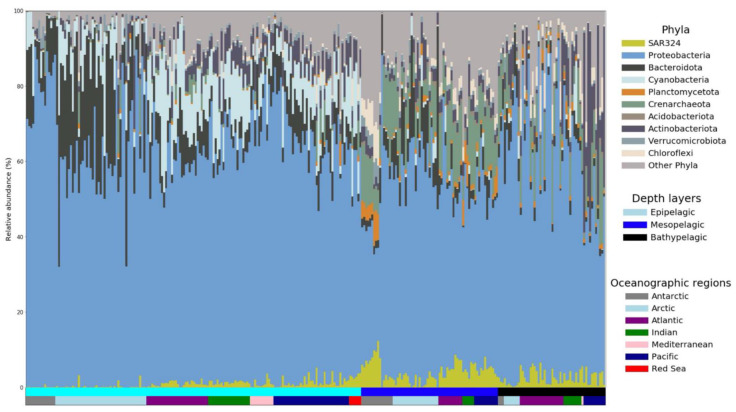
Relative sequence abundance of individual taxa of the microbial community in different oceanic basins based on annotated miTags. Each single bar on the *x*-axis represents a unique sample; they are ordered according to their depth layers from surface (**left** side) to bathypelagic (**right** side) and according to their geographic location within each depth layer.

SAR324 has been previously shown to be ubiquitous in the marine environment [17]. To obtain a more detailed estimate of the global abundance of SAR324 in epi-, meso- and bathypelagic waters, we accessed the publicly available data from the TARA Ocean and Malaspina expedition in addition to metagenomic datasets from polar environments [28] and from the water underneath the Ross Ice Shelf [30] to extract miTAGs belonging to SAR324 and calculate their relative 16S rRNA gene sequence abundance (Figure 1). SAR324 is ubiquitously present in almost all the sample locations. While its average relative sequence abundance in epipelagic samples is approximately 1%, its abundance increases in the bathypelagic and mesopelagic samples with an average of about 2.5% and 3.5%, respectively. In some cases, especially in extreme environments such as oxygen minimum zones, SAR324 can reach a relative sequence abundance of >20% [24,58]. Similarly high values (~15%) were observed in the oxygenated water column under the Ross Ice Shelf, suggesting that high relative sequence abundances of SAR324 are not restricted to low oxygen environments (Figure 1).

### 3.2. SAR324 Genomic Characteristics

To better understand the phylogenetic and functional diversity of SAR324 in the ocean and how it compares to other environments, we compiled and generated (>64% completeness) genomes of at least medium quality according to the minimum information about metagenome-assembled genome [59]. From a total number of 83 genomes from various sources, 25 passed our de-replication and quality filtering (Table 1, Supplementary Figure S1). We used a threshold of >98.5% average nucleotide identity (ANI) to exclude identical genomes and selected the most complete and least contaminated representative, a successful approach used in similar previous genomic studies (e.g., [60]). All the selected genomes are >64% in completeness and less than 3% contaminated, which are rather strict thresholds compared to those used in similar studies [60,61,62]. The GC-content of the genomes ranged between 39.1–57.4 (Table 1). Group 2B has a significantly higher GC-content when compared to the other groups with approximately 10% above the average with a GC-content of 57% (Student’s *t*-test *p* < 0.001, Table 1). A selection toward an increasing GC-content has been observed in some bacterial clusters, but the reasons remain unclear [63]. The average genome size of the SAR324 genomes was around 3.1 Mbp. The number of contigs varied between 84 and 1465. Coding density was evenly distributed with an average of 88%.

### 3.3. Global Phylogeny of SAR324

The identified SAR324 genomes were originally collected from a broad spectrum of locations (Supplementary Table S1). Some of the genomes obtained from the National Center for Biotechnology Information (NCBI) databases lack a detailed sample site description. Aside from varying depth layers in the open ocean (0–4000 m), the SAR324 genomes stem from a large variety of sites such as hydrothermal vents, a marine sponge (*Neamphius Huxleyi*) metagenome, a brine pool in the Red Sea and the seawater under 400 m of ice off Antarctica. Consistently, SAR324 has been found in diverse marine environments ranging from surface waters down to hydrothermal vents in the deep sea [22,58,64]. Surprisingly, three of the genomes were originally extracted from aquifers and one from estuarine sediments. Although these genomes are publicly available, to our knowledge, this is the first study investigating non-marine SAR324 genomes and comparing them to those originating from a marine environment.

**Figure 2:**
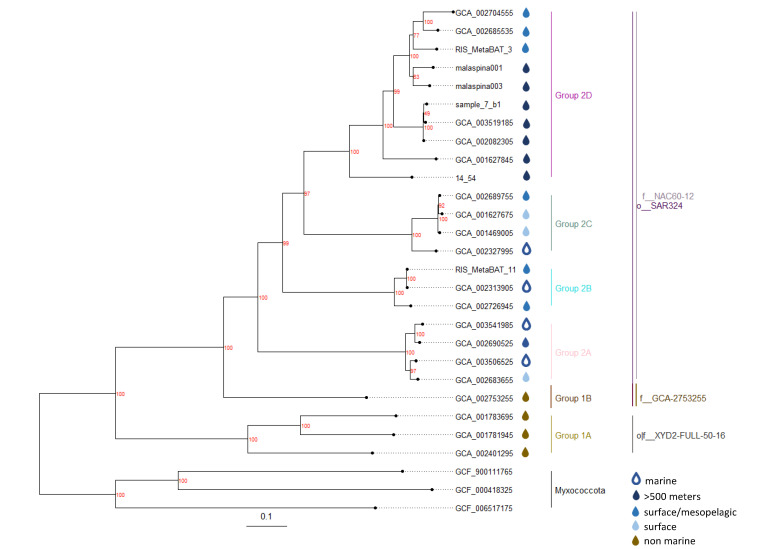
Phylogenetic maximum likelihood tree with de-replicated SAR324 genomes as well as genomes belonging to the phylum *Myxococcota* as an outgroup. Additionally, genomes are annotated with their origin based on the information available in public databases and their phylogeny based on the GTDB. More information regarding the sample location can be found in Supplementary Table S1. The tree is based on 120 bacterial marker genes and calculated with IQtree. We assigned names and colors to the different SAR24 clades based on their phylogeny and environment. The *x*-axis shows the phylogenetic distance scale.

We used HMM models to identify 120 bacterial genes that were then used to construct the phylogenetic tree of the SAR324 genomes (Figure 2). The phylogenetic clustering served to differentiate groups of SAR324 and mostly overlapped with the existing taxonomy established by the GTDB (See Methods and Figure 2). Two clusters resulted from aquifers and estuaries, and since they are the only genomes from non-marine environments and also different on at least the family-level from the remaining genomes according to the GTDB taxonomy, they were named Group 1A and 1B, respectively. Group 1A contains two genomes from terrestrial aquifers and one from an oxic subseafloor aquifer. Group 1B is made up of a single genome originating from estuarine sediments in Australia. The remaining genomes, all originating from marine sites, formed Group 2 and were further split up into the four subclades A, B, C and D. As we only have incomplete information regarding the origin of some genomes (especially clade 2B), it is difficult to assign environmental characteristics to the various subclades. However, based on the available sample site descriptions, clades 2A and 2C appear to be present in surface waters with no representatives in the deep sea. On the other hand, clade 2D almost exclusively contains sequences from the deep ocean members and extreme environments such as hydrothermal vents and brine pools (Table 1).

An additional phylogenetic assessment was performed with 16S rRNA sequences from nine of our SAR324 genomes. The clustering of the 16S rRNA sequences (Supplementary Figure S2) was mostly congruent to the previously established protein marker tree. 16S rRNA sequences extracted from genomes belonging to the same subclade in Group 2 are also assigned to the same OTU based on a 90% sequence similarity level in the Microbe Atlas Project (https://microbeatlas.org/, accessed on 20 November 2021), further demonstrating the robustness of the different clades and groups that were assigned within SAR324.

### 3.4. Metabolic Potential of SAR324

**Figure 3:**
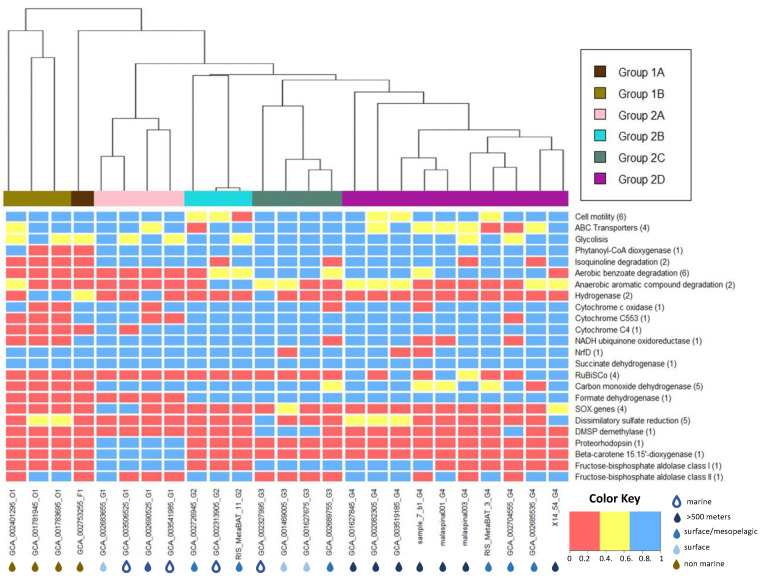
Presence/absence of selected genes or traits. The number in parenthesis indicates how many genes were used to define the respective trait (for more information regarding which genes were used, see Supplementary Table S2). Red color indicated the gene/trait is not present (contains <33% of the genes encoding the respective enzyme/pathway), yellow indicated partially present (33–66%) and blue present (>66%). On the bottom, genomes are additionally annotated with their respective environment.

To study the metabolic potential of the SAR324 phylum, we performed a pangenomic analysis, characterizing their functional core and accessory genes. Each phylogenetically generated group showed specific accessory genes not found in any of the other subclusters (Figure 3). Genes that were significantly different between the SAR324 clusters, main pathways (both heterotrophic and autotrophic), important proteins and enzymes investigated in previous studies on SAR324 as well as other bacterial lineages were examined in detail and can be found in Supplementary Table S2. The most important findings are summarized below.

#### 3.4.1. Energy Transport Chain

Terminal electron acceptors for respiration of SAR324 near hydrothermal vents have been discussed in great detail in earlier studies [22,23], but it is unknown in SAR324 from other environments. SAR324 appears to be capable of a flexible and diverse electron chain via NADH:ubiquinone oxidoreductase (complex I), succinate dehydrogenase (complex II), and as the last step of the respiratory chain, a high-affinity heme/copper-type cytochrome c oxidase (complex IV) was identified (Figure 3, Supplementary Table S2). However, in our analysis, neither the canonical nor an alternative complex III were found. A variety of metalloproteins (multi-heme C-type cytochromes), most prominently c553 and c4, responsible for different tasks in electron transfer were identified in most (22 out of 25) SAR324 bins. Our analysis showed that almost the entire electron acceptor chain [22] is not only present around hydrothermal vents but in all SAR324 cells in marine environments (Figure 3). However, cytochrome c oxidase is absent in the genomes from terrestrial aquifers belonging to Group 1, which could be a consequence of the hypoxic conditions in the terrestrial aquifer from which they were collected. In those genomes, Group 1d hydrogenases were present according to the HydDB, indicating the potential of hydrogenotrophic respiration [54].

#### 3.4.2. Central Carbon Metabolism

All 25 SAR324 genomes possess the most important genes encoding proteins for basic heterotrophic pathways such as glycolysis and the tricarboxylic acid (TCA) cycle (Figure 3, Supplementary Table S2). In addition, genes for the breakdown of fatty acids via the β-oxidation pathway, such as acyl-CoA dehydrogenase and acetyl-CoA C-acetyltransferase and alcohol degradation, were also identified in all genomes (Supplementary Table S2). In contrast, the ability to degrade complex aromatic carbohydrates was only widespread in the marine SAR324 Group 2. While aerobic benzoate degradation is restricted to groups 2C and 2D, all SAR324 genomes belonging to Group 2 appear to have the capability of degrading isoquinoline. Genes encoding phenylacetic acid degradation proteins *PaaD* and *PaaI* associated with the anaerobic benzoate degradation were found in genomes belonging to Group 1B, 2B, 2C and 2D (Figure 3).

Previous studies have suggested that some SAR324 strains are able to fix carbon via the Calvin–Benson–Bassham (CBB) cycle or the Wood–Ljungdahl pathway [22,23,61]. However, we could only verify the presence of the CBB pathway. Five genomes, all belonging to Group 2D, contained genes encoding the key enzymes for the CBB cycle, e.g., small and large subunits of RuBisCO and other essential proteins for the autotrophic carbon fixation, such as the RuBisCO activation proteins CbbQ and CbbO (Figure 3). Although most of the genes for the Wood–Ljungdahl pathway were present in Groups 2C and 2D, the key enzyme CO-methylating acetyl-CoA synthase was missing in all genomes. Genes encoding propionyl-CoA carboxylase carboxyl transferase, an enzyme that may be responsible for the anaplerotic incorporation of bicarbonate into the TCA cycle, were detected in most genomes from Groups 2B, 2C and 2D. Other pathways for autotrophic CO2 fixation/assimilation (3-HP/malyl-CoA cycle, reverse Krebs cycle) were not found.

Furthermore, while some more genes associated with C1 metabolism were present in groups 2B, 2C and 2D (e.g., formate dehydrogenase, formate-tetrahydrofolate ligase), we did not identify any particulate methane monooxygenase (*pMMO*) or methanol dehydrogenase genes, suggesting that the majority of SAR324 cells are not able to utilize methane. However, genomes from groups 2B, 2C and 2D can potentially metabolize other reduced one-carbon compounds: e.g., four genomes (of which three belong to Group 2B and one to Group 2D) contain genes encoding a dimethylsulfoniopropionate (DMSP) demethylase indicating that some SAR324 can utilize parts of the C1 pathway for DMSP degradation [22].

Additionally, most subunits of the carbon monoxide dehydrogenase complex (CODH, *cox* genes) were found in all marine SAR324 genomes (Figure 3). Its presence was previously shown in SAR324 living close to the South mid-Atlantic ridge hydrothermal vents, where it was assumed that the *cox* genes in SAR324 played a role in the reductive acetyl-CoA pathway [23]. However, due to the absence of acetyl-CoA synthase in all our bins, we propose another potential use of carbon monoxide dehydrogenase. Recent studies suggest that carbon monoxide oxidation is an important energy supplement, highly abundant in the deep ocean [65,66]. Thus, it is very likely that the *cox* genes in SAR324 are an alternative way of gaining energy from carbon monoxide and support mixotrophic growth in oligotrophic conditions.

#### 3.4.3. Role of Nitrogen and Sulfur Compounds in SAR324

Evidence of a nitrite reductase (*nirK*) copper-containing form was found previously in SAGs and small contigs with high similarity to the SAG genes around hydrothermal vents [21,22]. However, no *nirK* genes were detected in any genomes in this study (Supplementary Table S2). This leads us to support the claim of Cao et al. (2016) that those genes might be the result of phage islands and nitrogen compounds play no or only a minor role as electron acceptors.

The reduction and oxidation of sulfuric compounds have been assumed to be important parts of the metabolism of SAR324 [21]. Our annotations suggest that the reverse dissimilatory sulfate reduction pathway is not a common feature of SAR324 but perhaps an isolated trait of SAR324 strains living in anoxic environments. The sulfate reduction genes sulfate adenylyltransferase (*sat*) and dissimilatory sulfite reductase (*dsr*) were present in two bins belonging to the basal Group 1 found in a terrestrial aquifer. Adenosine-5’-phosphosulfate reductase was not detected in those bins but is present in GCA_003519185 (from a deep-sea sponge metagenome), GCA_002082305 (from hydrothermal vents), 14_54 (from a Red Sea brine pool), GCA_001627845 (from a deep Red Sea sample close to hydrothermal activity) and GCA_002327995 (no origin information except “marine”). It cannot be excluded that SAR324 does not have the ability to perform dissimilatory sulfate reduction at all since *dsr* genes can perform an alternative function in species oxidizing sulfur via reversal of the sulfate reduction pathway [67]. Sulfur oxidation (*Sox*) genes responsible for the oxidation of thiosulfate [68] were only present in two genomes from Group 2A (Figure 3). Collectively, we conclude that sulfur-related pathways only play a role in the metabolism of SAR324 in very specific environments and are not a universal feature of the SAR324 cluster.

#### 3.4.4. Additional Traits

Genes encoding flagellar motility and cell adhesion were found in all genomes (Figure 3). Group 2B appears to have only half of the motility genes when compared to the other groups, potentially indicating that Group 2B is less relying on active movement toward nutrient sources and particle attachment.

The presence of various ATP-binding cassette (ABC-type) transporters for lipopolysaccharides, amino acids, oligopeptides and more indicates a heterotrophic or mixotrophic lifestyle in all SAR324 clades. Group 2D shows only approximately 60% of the ABC transporters when compared to other SAR324 groups, suggesting that they rely at least partially on the previously discussed autotrophic carbon fixation via the CBB cycle.

The presence of the gene encoding proteorhodopsin is an interesting feature of SAR324 observed exclusively in all Group 2A bins. Proteorhodopsin in SAR324 is believed to be a blue and green light-absorbing proton pump capable of generating a proton motive force across the cell membrane [69]. Its transcription in SAR324 was recently observed in the North Pacific subtropical gyre [69]. Group 2A also possesses genes encoding 15, 15’-β-carotene dioxygenase (*blh*) (Figure 2, Supplementary Table S2). *Blh* genes are responsible for the production of retinal via the carotenoid biosynthetic pathway, and retinal is essential for the functionality of proteorhodopsin [70]. This photoheterotrophic capability might provide SAR324 Group 2A with a competitive advantage in the oligotrophic ocean since proteorhodopsin has been demonstrated to promote the survival of bacteria, for instance, during starvation and/or oligotrophic conditions [71,72].

### 3.5. Global Ocean Metatranscriptomic Analysis of SAR324

**Figure 4:**
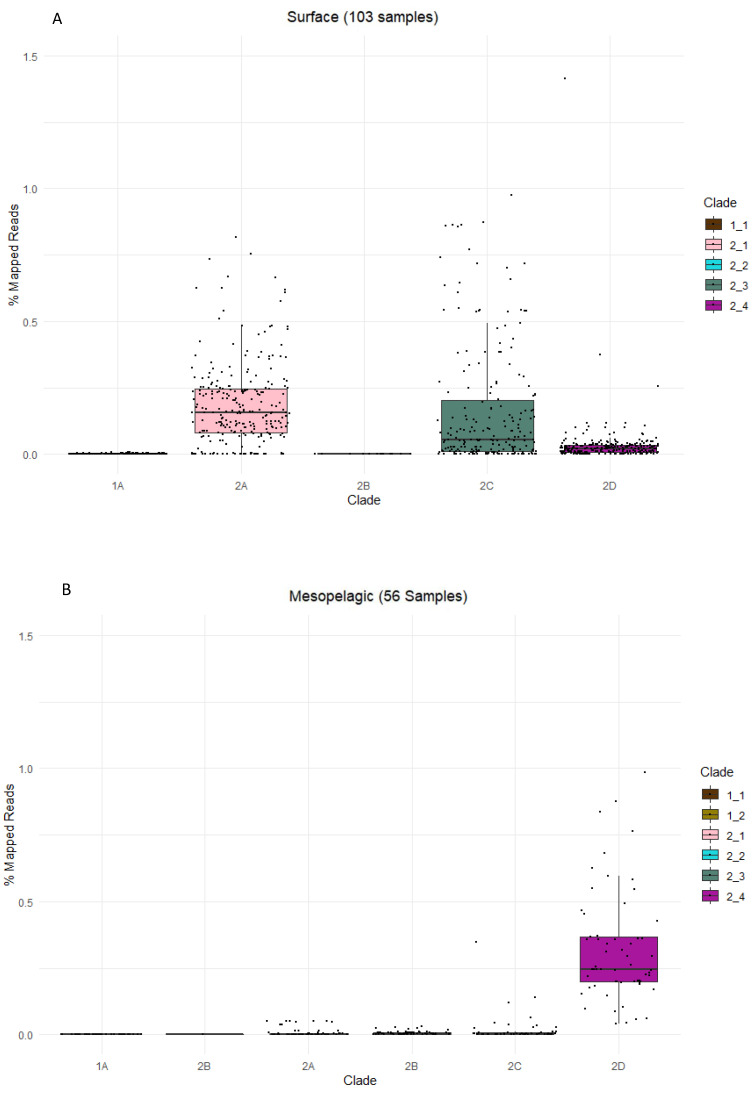
Bar plots showing the percentage of metatranscriptomic reads that mapped to the respective SAR324 clades relative to the total number of reads present in each sample on the *x*-axis for Surface (**A**) and Mesopelagic (**B**). The largest number of SAR324 mapped reads in the surface layer belong to clades 2A and 2C, while 2D appears to be the dominant clade in the mesopelagic region.

A metagenomic approach provides valuable insights into the metabolic potential. To go a step further and gain insights into the actual activity of SAR324 in the global ocean, we used the recently released metatranscriptomic reads from the TARA Ocean cruise [73]. As expected, we did not see any substantial number of genes expressed belonging to the SAR324 Group 1 in our global ocean library, supporting the hypothesis that they are restricted to non-marine environments. Additionally, we observed that both Group 2A and 2C appear to be active in the surface waters, while Group 2D was the only clade expressing its genes in the mesopelagic realm (Figure 4). In the mixed layer as well as the deep chlorophyll maximum (DCM), Group 2D is the most expressed, while Group 2A and 2C are active as well (Supplementary Figure S3). Group 2B appears not to be active in the limited range of environments covered by the TARA Ocean expedition, suggesting that they are found outside of the mesopelagic and surface layers.

### 3.6. Recent Insights into SAR324 at the ALOHA Station off Hawaii

Recently, a paper with a similar approach has been published [25]. SAR324 genomes from different depths at the ALOHA station off Hawaii have been reconstructed, and additionally, genomes from public databases have been mapped to them to create population genomes. Instead of combining similar genomes as carried out by Boeuf et al. (2021), we chose to use only the best genome (based on checkM and dRep) to represent a cluster, accepting a slight decrease in observed genes for a lower potential of contamination.

Additionally, Boeuf et al. (2021) also split up the genomes into different clusters based on their ANI, resulting in five different clusters, which mostly corresponded to four ecotypes (surface-only, above deep chlorophyll maximum (DCM), below DCM, deep), resulting from the abundance of the different SAR324 types in different depth layers. Those clusters most likely correspond to subclades of what we call Group 2 SAR324 since their study did not include any non-marine SAR324 genomes (Group 1). For parts of their analysis, they used the same genomes as presented in our research, thus making it possible to link the different subclades. Their subclade A corresponds to our subclade 2A (GCA002690525), their subclade B corresponds to our subclade 2C (GCA001469005), their subclade D and E correspond to our subclade 2D (GCA001627845 and GCA001781945 respectively), whereas our subclade 2B probably corresponds to their outlier with a significantly higher GC content. Similar to our work, Boeuf et al. (2021) showed a depth stratification of the different SAR324 clades and different metabolic potential depending on their preferred habitat.

We confirm some of the observations of Boeuf et al. (2021), such as the presence of proteorhodopsin in the surface clade 2A and the CBB cycle in the deep ocean clade 2D on a global scale, and demonstrate that most of their other observations are not confined to the ALOHA station in the North Pacific Gyre but are globally applicable.

## 4. Conclusions

This is the first pangenomic study of SAR324 genomes covering all potential environments where they live in. As such, it provides unprecedented insights into the phylogeny, metabolism and ecology of this phylum in the global ocean. We found that SAR324 is not only present in the marine environment but also in terrestrial aquifers and estuaries. We propose different SAR324 subclusters supported by the phylogenetic analyses, splitting up the SAR324 phylum into two main clusters: Groups 1 and 2.

Group 1 consists of genomes from aquifers and estuarine sediments. Group 2 consists solely of bins originating from diverse marine locations. Group 1 seems to be a mobile, mostly heterotrophic clade with the *Bdellovibrionate* and *Myxococcales* as neighboring phyla. In Group 2, new adaptations and lateral gene transfer in the marine environment are likely causes for a variety of newly acquired pathways to generate energy and survive oligotrophic conditions. For instance, phytanoyl-CoA dioxygenase, an enzyme degrading the lipid chain in chlorophyll a [74], only appeared in oceanic genomes, hence suggesting that the degradation of sinking photosynthetic biomass in marine SAR324 might be one of the first adaptations to the new marine environment. Still, each Group 2 subclade evolved its own specialties and developed specific adaptations to its respective environment. For example, Group 2A possesses genes encoding proteorhodopsin, 15,15’ -β-carotene dioxygenase (*blh*) and various types of ABC-type transporters and heterotrophic pathways, which indicates a photoheterotrophic clade present in surface waters, congruent to the information we have regarding the origin of those genomes (Table 1). In turn, the deep-sea affiliated Group 2D is more associated with aromatic compound degradation and an autotrophic way of carbon fixation via the CBB cycle as well as anaplerotic incorporation of bicarbonate into the TCA cycle.

Collectively, the SAR324 phylum exhibits a remarkable metabolic versatility, potentially explaining its widespread distribution and relevance in the ocean. The presence of a high-affinity cytochrome c oxidase with no trace of a low-affinity oxidase, together with the potential for sulfate reduction in selected environments as well as genes for both oxic and anoxic degradation of aromatic compounds supports the general observation that SAR324 is highly abundant especially (but not only) in low oxygen zones [58,75,76]. In the generally oxygenated water column, such low-oxygen conditions might be found in detrital particles such as marine snow, consistent with the suggestion that SAR324 has a preferential particle-attached life mode in the deep ocean [21]. Additionally, by mapping the metatranscriptomic TARA Ocean dataset to our genomic information, we can identify different clades found in the surface vs. deep waters. Collectively, SAR324 shows the potential to play a key role in global biogeochemical cycles.

## Figures and Tables

**Table 1:** Genome properties of all the genomes which passed our quality filtering and de-replication (see Material and Methods for details). All genomes are ordered according to their completeness values. * An asterisk next to a genome name indicates a bin containing more than one copy of the genome. # In the columns is an abbreviation for “Numbers of”. The origin of genomes which did not pass our quality filters is shown in Supplementary Table S1.

ID	Environment	Completeness	Contamination	Genome Size (Mbp)	GC %	# Contigs	N50 Contigs (KBP)	Coding Density %
GCA_001469005	Red Sea water column Station 34–depth 10 m	89.09	0.0	3.49	47.1	312	16.27	90
GCA_001627675	Red Sea water column Station 34–depth 25 m	72.17	2.94	2.54	47.46	440	6.54	91
GCA_001627845	Red Sea water column Station 192–depth 500 m	89.54	0.0	3.17	42.89	90	58.32	89
GCA_001781945	Rifle well CD01 at 16ft depth; 0.1 μm filter at time point D, USA: Rifle, CO	91.96	1.68	3.12	56.51	84	65.77	91
GCA_001783695	Rifle well CD01 at 16ft depth; 0.1 μm filter at time point D, USA: Rifle, CO	91.83	1.68	3.14	49.59	149	37.4	91
GCA_002082305	hydrothermal plumes on the South Mid-Atlantic Ridge	90.35	0.0	2.8	42.3	148	26.08	89
GCA_002313905	marine	81.27	0.84	3.15	55.43	595	8.63	88
GCA_002327995	marine	87.04	0.84	4.04	45.73	470	14.94	86
GCA_002401295	Atlantic Ocean: North Pond, oxic subseafloor aquifer	69.22	2.95	3.08	41.71	182	23.04	87
GCA_002683655	Chile-Peru Current Coastal Province, 5–1000 m (TARA)	80.16	2.33	3.15	46.19	109	42.34	89
GCA_002685535 *	Chile-Peru Current Coastal Province, 5–1000 m (TARA)	87.85	2.75	7.29	46.09	245	51.04	77
GCA_002689755	Mediterranean Sea, 5–1000 m (TARA)	65.12	0.85	2.05	46.5	161	12.88	92
GCA_002690525	Mediterranean Sea, 5–1000 m (TARA)	80.24	1.68	3.12	44.81	187	18.62	88
GCA_002704555	Mediterranean Sea, 5–1000 m (TARA)	69.42	1.05	1.97	38.89	114	19.51	89
GCA_002726945	North Pacific Ocean, 5–1000 m (TARA)	89.88	2.2	2.6	57.34	156	18.45	89
GCA_002753255 *	Australia: Punkally Creek Sediment	78.03	0.96	5.06	41.83	530	14.91	90
GCA_003506525	marine	72.8	0.95	3.48	44.67	720	7.44	83
GCA_003519185	Neamphius huxleyi metagenome	68.15	0.0	2.13	40.87	1465	2.25	73
GCA_003541985	marine	67.36	0.84	2.48	44.62	592	5.66	83
14_54	Red Sea brine pool	87.34	0.17	3.45	43.43	374	12.52	87
RIS_MetaBAT_11	Ross Ice Shelf Antarctica	79.08	0.89	2.47	57.09	297	10.42	92
RIS_MetaBAT_3	Ross Ice Shelf Antarctica	81.27	1.01	1.93	40.12	285	8.09	90
malaspina001	Malaspina Deep Sea Samples	91.15	2.69	2.61	42.46	284	20.73	89
malaspina003	Malaspina Deep Sea Samples	91.26	0.63	2.77	42.98	222	14.14	90
sample_7_b1	45° N, 178° E, Deep Sea	65.19	1.58	1.8	40.91	1138	1.57	88

## Data Availability

The raw data supporting the conclusions of this article will be made available by the authors, without undue reservation to any qualified researcher.

## Supplementary Materials

The following supporting information can be downloaded at: https://www.mdpi.com/article/10.3390/biology11040599/s1, Figure S1: Dereplication process; Figure S2: 16s rRNA Phylogenetic Tree; Figure S3: Metatranscriptomic read mapping in the DCM and mixed layer; Table S1: Properties and origin of all analyzed genomes; Table S2: Presence/absence matrix of selected genes.

## References

[1] Azam F., Fenchel T., Field J., Gray J., Meyer-Reil L., Thingstad F. (1983). The Ecological Role of Water-Column Microbes in the Sea. Mar. Ecol. Prog. Ser..

[2] Herndl G.J., Reinthaler T. (2013). Microbial control of the dark end of the biological pump. Nat. Geosci..

[3] Partensky F., Hess W.R., Vaulot D. (1999). Prochlorococcus, a Marine Photosynthetic Prokaryote of Global Significance. Microbiol. Mol. Biol. Rev..

[4] Johnson Z.I., Zinser E.R., Coe A., McNulty N.P., Woodward E.M.S., Chisholm S.W. (2006). Niche Partitioning Among *Prochlorococcus* Ecotypes Along Ocean-Scale Environmental Gradients. Science.

[5] Newton R.J., Griffin L.E., Bowles K.M., Meile C., Gifford S., Givens C.E., Howard E.C., King E., Oakley C.A., Reisch C.R. (2010). Genome characteristics of a generalist marine bacterial lineage. ISME J..

[6] Shiba T. (1991). Roseobacter litoralis gen. nov., sp. nov., and Roseobacter denitrificans sp. nov., Aerobic Pink-Pigmented Bacteria which Contain Bacteriochlorophyll a. Syst. Appl. Microbiol..

[7] Morris R.M., Rappé M.S., Connon S.A., Vergin K., Siebold W.A., Carlson C.A., Giovannoni S.J. (2002). SAR11 clade dominates ocean surface bacterioplankton communities. Nature.

[8] Herndl G., Agogué H., Baltar F., Reinthaler T., Sintes E., Varela M. (2008). Regulation of aquatic microbial processes: The ‘microbial loop’ of the sunlit surface waters and the dark ocean dissected. Aquat. Microb. Ecol..

[9] Sharma R., Ranjan R., Kapardar R.K. (2022). “Unculturable” bacterial diversity: An untapped resource. Microb. Divers..

[10] Sogin M.L., Morrison H.G., Huber J.A., Welch D.M., Huse S.M., Neal P.R., Arrieta J.M., Herndl G.J. (2006). Microbial diversity in the deep sea and the underexplored “rare biosphere”. Proc. Natl. Acad. Sci. USA.

[11] Danovaro R., Snelgrove P.V.R., Tyler P. (2014). Challenging the paradigms of deep-sea ecology. Trends Ecol. Evol..

[12] Streit W.R., Schmitz R.A. (2004). Metagenomics—The key to the uncultured microbes. Curr. Opin. Microbiol..

[13] Caporaso J.G., Lauber C.L., Walters W.A., Berg-Lyons D., Huntley J., Fierer N., Owens S.M., Betley J., Fraser L., Bauer M. (2012). Ultra-high-throughput microbial community analysis on the Illumina HiSeq and MiSeq platforms. ISME J..

[14] Baltar F., Bayer B., Bednarsek N., Deppeler S., Escribano R., Gonzalez C.E., Hansman R.L., Mishra R.K., Moran M.A., Repeta D.J. (2019). Towards Integrating Evolution, Metabolism, and Climate Change Studies of Marine Ecosystems. Trends Ecol. Evol..

[15] Boetius A. (2019). Global change microbiology—Big questions about small life for our future. Nat. Rev. Microbiol..

[16] Hutchins D.A., Jansson J.K., Remais J.V., Rich V.I., Singh B.K., Trivedi P. (2019). Climate change microbiology—Problems and perspectives. Nat. Rev. Microbiol..

[17] Pommier T., Pinhassi J., Hagström Å. (2005). Biogeographic analysis of ribosomal RNA clusters from marine bacterioplankton. Aquat. Microb. Ecol..

[18] Wright T.D., Vergin K.L., Boyd P.W., Giovannoni S.J. (1997). A Novel Delta-Subdivision Proteobacterial Lineage from the Lower Ocean Surface Layer. Appl. Environ. Microbiol..

[19] Salazar G., Cornejo-Castillo F.M., Benítez-Barrios V., Fraile-Nuez E., Álvarez-Salgado X.A., Duarte C.M., Gasol J.M., Acinas S.G. (2016). Global diversity and biogeography of deep-sea pelagic prokaryotes. ISME J..

[20] Parks D.H., Chuvochina M., Waite D.W., Rinke C., Skarshewski A., Chaumeil P.-A., Hugenholtz P. (2018). A standardized bacterial taxonomy based on genome phylogeny substantially revises the tree of life. Nat. Biotechnol..

[21] Swan B.K., Martinez-Garcia M., Preston C.M., Sczyrba A., Woyke T., Lamy D., Reinthaler T., Poulton N.J., Masland E.D.P., Gomez M.L. (2011). Potential for Chemolithoautotrophy Among Ubiquitous Bacteria Lineages in the Dark Ocean. Science.

[22] Sheik C.S., Jain S., Dick G.J. (2014). Metabolic flexibility of enigmatic SAR324 revealed through metagenomics and metatranscriptomics: Disentangling the ecophysiological role of SAR324. Environ. Microbiol..

[23] Cao H., Dong C., Bougouffa S., Li J., Zhang W., Shao Z., Bajic V.B., Qian P.-Y. (2016). Delta-proteobacterial SAR324 group in hydrothermal plumes on the South Mid-Atlantic Ridge. Sci. Rep..

[24] Quero G.M., Celussi M. (2019). Inorganic and Organic Carbon Uptake Processes and Their Connection to Microbial Diversity in Meso- and Bathypelagic Arctic Waters (Eastern Fram Strait). Microb. Ecol..

[25] Boeuf D., Eppley J.M., Mende D.R., Malmstrom R.R., Woyke T., DeLong E.F. (2021). Metapangenomics reveals depth-dependent shifts in metabolic potential for the ubiquitous marine bacterial SAR324 lineage. Microbiome.

[26] Sunagawa S., Coelho L.P., Chaffron S., Kultima J.R., Labadie K., Salazar G., Djahanschiri B., Zeller G., Mende D.R., Alberti A. (2015). Structure and function of the global ocean microbiome. Science.

[27] Duarte C.M. (2015). Seafaring in the 21St Century: The Malaspina 2010 Circumnavigation Expedition. Limnol. Oceanogr. Bull..

[28] Cao S., Zhang W., Ding W., Wang M., Fan S., Yang B., Mcminn A., Wang M., Xie B., Qin Q.-L. (2020). Structure and function of the Arctic and Antarctic marine microbiota as revealed by metagenomics. Microbiome.

[29] Salazar G., Paoli L., Alberti A., Huerta-Cepas J., Ruscheweyh H.-J., Cuenca M., Field C.M., Coelho L.P., Cruaud C., Engelen S. (2019). Gene Expression Changes and Community Turnover Differentially Shape the Global Ocean Metatranscriptome. Cell.

[30] Martínez-Pérez C., Greening C., Bay S.K., Lappan R.J., Zhao Z., De Corte D., Hulbe C., Ohneiser C., Stevens C., Thomson B. (2022). Phylogenetically and functionally diverse microorganisms reside under the Ross Ice Shelf. Nat. Commun..

[31] Logares R., Sunagawa S., Salazar G., Cornejo-Castillo F.M., Ferrera I., Sarmento H., Hingamp P., Ogata H., de Vargas C., Lima-Mendez G. (2014). Metagenomic 16S rDNA Illumina tags are a powerful alternative to amplicon sequencing to explore diversity and structure of microbial communities: Using *mi tag* s to explore microbial communities. Environ. Microbiol..

[32] Quast C., Pruesse E., Yilmaz P., Gerken J., Schweer T., Yarza P., Peplies J., Glöckner F.O. (2012). The SILVA ribosomal RNA gene database project: Improved data processing and web-based tools. Nucleic Acids Res..

[33] Gruber-Vodicka H.R., Seah B.K.B., Pruesse E. (2020). PhyloFlash: Rapid Small-Subunit RRNA Profiling and Targeted Assembly from Metagenomes. mSystems.

[34] Wickham H. (2011). ggplot2. WIREs Comp. Stat..

[35] Andrews S. (2010). FastQC. Babraham Bioinforma.

[36] Bankevich A., Nurk S., Antipov D., Gurevich A.A., Dvorkin M., Kulikov A.S., Lesin V.M., Nikolenko S.I., Pham S., Prjibelski A.D. (2012). SPAdes: A New Genome Assembly Algorithm and Its Applications to Single-Cell Sequencing. J. Comput. Biol..

[37] Li D., Luo R., Liu C.-M., Leung C.-M., Ting H.-F., Sadakane K., Yamashita H., Lam T.-W. (2016). MEGAHIT v1.0: A fast and scalable metagenome assembler driven by advanced methodologies and community practices. Methods.

[38] Strous M., Kraft B., Bisdorf R., Tegetmeyer H.E. (2012). The Binning of Metagenomic Contigs for Microbial Physiology of Mixed Cultures. Front. Microbio..

[39] Kang D.D., Froula J., Egan R., Wang Z. (2015). MetaBAT, an efficient tool for accurately reconstructing single genomes from complex microbial communities. PeerJ.

[40] Wu Y.-W., Simmons B.A., Singer S.W. (2016). MaxBin 2.0: An automated binning algorithm to recover genomes from multiple metagenomic datasets. Bioinformatics.

[41] Sieber C.M.K., Probst A.J., Sharrar A., Thomas B.C., Hess M., Tringe S.G., Banfield J.F. (2018). Recovery of genomes from metagenomes via a dereplication, aggregation and scoring strategy. Nat. Microbiol..

[42] Parks D.H., Imelfort M., Skennerton C.T., Hugenholtz P., Tyson G.W. (2015). CheckM: Assessing the quality of microbial genomes recovered from isolates, single cells, and metagenomes. Genome Res..

[43] Eren A.M., Esen Ö.C., Quince C., Vineis J.H., Morrison H.G., Sogin M.L., Delmont T.O. (2015). Anvi’o: An advanced analysis and visualization platform for ‘omics data. PeerJ.

[44] Buchfink B., Xie C., Huson D.H. (2015). Fast and sensitive protein alignment using DIAMOND. Nat. Methods.

[45] Zhao Z., Baltar F., Herndl G.J. (2020). Linking extracellular enzymes to phylogeny indicates a predominantly particle-associated lifestyle of deep-sea prokaryotes. Sci. Adv..

[46] Olm M.R., Brown C.T., Brooks B., Banfield J.F. (2017). dRep: A Tool for Fast and Accurate Genome De-Replication That Enables Tracking of Microbial Genotypes and Improved Genome Recovery from Metagenomes. Bioinformatics.

[47] Huang Y., Gilna P., Li W. (2009). Identification of ribosomal RNA genes in metagenomic fragments. Bioinformatics.

[48] Nguyen L.-T., Schmidt H.A., von Haeseler A., Minh B.Q. (2015). IQ-TREE: A Fast and Effective Stochastic Algorithm for Estimating Maximum-Likelihood Phylogenies. Mol. Biol. Evol..

[49] Hoang D.T., Chernomor O., von Haeseler A., Minh B.Q., Vinh L.S. (2018). UFBoot2: Improving the Ultrafast Bootstrap Approximation. Mol. Biol. Evol..

[50] Yu G., Smith D.K., Zhu H., Guan Y., Lam T.T. (2017). ggtree: An R package for visualization and annotation of phylogenetic trees with their covariates and other associated data. Methods Ecol. Evol..

[51] Hyatt D., Chen G.L., LoCascio P.F., Land M.L., Larimer F.W., Hauser L.J. (2010). Prodigal: Prokaryotic gene recognition and translation initiation site identification. BMC Bioinform..

[52] Aziz R.K., Bartels D., Best A., DeJongh M., Disz T., Edwards R.A., Formsma K., Gerdes S., Glass E.M., Kubal M. (2008). The RAST Server: Rapid annotations using subsystems technology. BMC Genom..

[53] Quevillon E., Silventoinen V., Pillai S., Harte N., Mulder N., Apweiler R., Lopez R. (2005). InterProScan: Protein domains identifier. Nucleic Acids Res..

[54] Søndergaard D., Pedersen C.N.S., Greening C. (2016). HydDB: A web tool for hydrogenase classification and analysis. Sci. Rep..

[55] Delmont T.O., Eren E.M. (2018). Linking pangenomes and metagenomes: The Prochlorococcus metapangenome. PeerJ.

[56] Li H., Durbin R. (2009). Fast and accurate short read alignment with Burrows-Wheeler transform. Bioinformatics.

[57] Bushnell B. (2014). BBMap: A Fast, Accurate, Splice-Aware Aligner.

[58] Wright J.J., Konwar K.M., Hallam S.J. (2012). Microbial ecology of expanding oxygen minimum zones. Nat. Rev. Microbiol..

[59] Bowers R.M., Kyrpides N.C., Stepanauskas R., Harmon-Smith M., Doud D., Reddy T.B.K., Schulz F., Jarett J., Rivers A.R., Eloe-Fadrosh E.A. (2017). Minimum information about a single amplified genome (MISAG) and a metagenome-assembled genome (MIMAG) of bacteria and archaea. Nat. Biotechnol..

[60] Tully B.J. (2019). Metabolic diversity within the globally abundant Marine Group II Euryarchaea offers insight into ecological patterns. Nat. Commun..

[61] Cameron T.J., Temperton B., Swan B.K., Landry Z.C., Woyke T., Delong E.F., Stepanauskas R., Giovannoni S.J. (2014). Single-cell enabled comparative genomics of a deep ocean SAR11 bathytype. ISME J..

[62] Mehrshad M., Rodriguez-Valera F., Amoozegar M.A., López-García P., Ghai R. (2018). The enigmatic SAR202 cluster up close: Shedding light on a globally distributed dark ocean lineage involved in sulfur cycling. ISME J..

[63] Hildebrand F., Meyer A., Eyre-Walker A. (2010). Evidence of selection upon genomic GC-content in bacteria. PLoS Genet..

[64] Chitsaz H., Yee-Greenbaum J.L., Tesler G., Lombardo M.-J., Dupont C.L., Badger J.H., Novotny M., Rusch D.B., Fraser L.J., Gormley N.A. (2011). Efficient de novo assembly of single-cell bacterial genomes from short-read data sets. Nat. Biotechnol..

[65] Acinas S.G., Sánchez P., Salazar G., Cornejo-Castillo F.M., Sebastián M., Logares R., Sunagawa S., Hingamp P., Ogata H., Lima-Mendez G. (2019). Metabolic Architecture of the Deep Ocean Microbiome. Microbiology.

[66] Cordero P.R.F., Bayly K., Man Leung P., Huang C., Islam Z.F., Schittenhelm R.B., King G.M., Greening C. (2019). Atmospheric carbon monoxide oxidation is a widespread mechanism supporting microbial survival. ISME J..

[67] Thorup C., Schramm A., Findlay A.J., Finster K.W., Schreiber L. (2017). Disguised as a Sulfate Reducer: Growth of the Deltaproteobacterium Desulfurivibrio alkaliphilus by Sulfide Oxidation with Nitrate. mBio.

[68] Berben T., Overmars L., Sorokin D.Y., Muyzer G. (2019). Diversity and distribution of sulfur oxidation-related genes in thioalkalivibrio, a genus of chemolithoautotrophic and haloalkaliphilic sulfur-oxidizing bacteria. Front. Microbiol..

[69] Olson D.K., Yoshizawa S., Boeuf D., Iwasaki W., DeLong E.F. (2018). Proteorhodopsin variability and distribution in the North Pacific Subtropical Gyre. ISME J..

[70] Sabehi G., Loy A., Jung K.H., Partha R., Spudich J.L., Isaacson T., Hirschberg J., Wagner M., Béjà O. (2005). New insights into metabolic properties of marine bacteria encoding proteorhodopsins. PLoS Biol..

[71] Gómez-Consarnau L., Akram N., Lindell K., Pedersen A., Neutze R., Milton D.L., González J.M., Pinhassi J. (2010). Proteorhodopsin Phototrophy Promotes Survival of Marine Bacteria during Starvation. PLoS Biol..

[72] Palovaara J., Akram N., Baltar F., Bunse C., Forsberg J., Pedrós-Alió C., González J.M., Pinhassi J. (2014). Stimulation of growth by proteorhodopsin phototrophy involves regulation of central metabolic pathways in marine planktonic bacteria. Proc. Natl. Acad. Sci. USA.

[73] Sunagawa S., Acinas S.G., Bork P., Bowler C., Eveillard D., Gorsky G., Guidi L., Iudicone D., Karsenti E., Tara Oceans Coordinators (2020). Tara Oceans: Towards global ocean ecosystems biology. Nat. Rev. Microbiol..

[74] Schofield C.J., McDonough M.A. (2007). Structural and mechanistic studies on the peroxisomal oxygenase phytanoyl-CoA 2-hydroxylase (PhyH). Biochem. Soc. Trans..

[75] Fuchsman C.A., Kirkpatrick J.B., Brazelton W.J., Murray J.W., Staley J.T. (2011). Metabolic strategies of free-living and aggregate-associated bacterial communities inferred from biologic and chemical profiles in the Black Sea suboxic zone. FEMS Microbiol. Ecol..

[76] Carolan M.T. (2014). Quantifying Distributions of and Modeling Interactions among Sulfur- and Nitrogen- Cycling Chemolithoautotrophs in the Largest Oxygen Minimum Zone of the Global Ocean. Ph.D. Thesis.

