# Supplementary material for: Phylogeny and Metabolic Potential of the Candidate Phylum SAR324
Source: Biology (Basel). 2022 Apr 14;11(4):599. doi: 10.3390/biology11040599 (PMC9031357; doi:10.3390/biology11040599)
Supplement: Supplementary file 1 [file biology-11-00599-s001.zip › biology-1659073-supplementary.pdf]

Supplementary Information:

Phylogeny and metabolic potential of the phylum SAR324

Lukas Malfertheiner[1], Clara Martínez-Pérez[1,2], Zihao Zhao[1], Gerhard J. Herndl[1,3], Federico Baltar[1*]

[1] Department of Functional and Evolutionary Ecology, University of Vienna, 1090 Vienna, Austria

[2] Institute for Environmental Engineering, Department of Civil, Environmental and Geomatic Engineering, Eidgenössische Technische Hochschule (ETH) Zürich, 8093 Zurich, Switzerland

[3] NIOZ, Department of Marine Microbiology and Biogeochemistry, Royal Netherlands Institute for Sea Research, Utrecht University, 1790 AB Den Burg, Texel, The Netherlands

Contains:

- Figure S1-S3

- Tables S1-S2

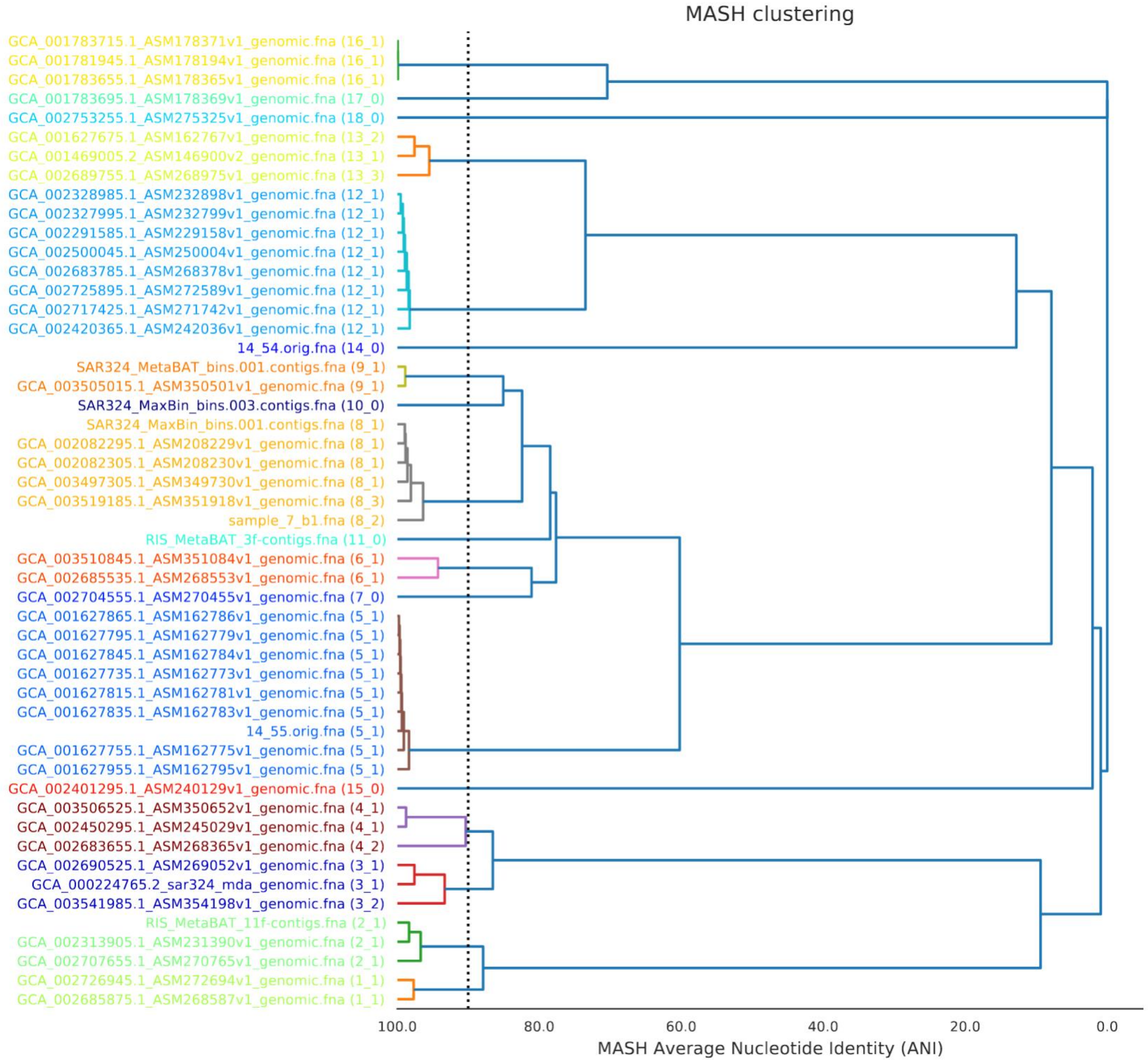

Figure S1. Dereplication process. Out of very similar genomes, the best representative was chosen based mostly on completion and contamination percentages (see dRep score, Supplementary Table 1) for downstream analysis.

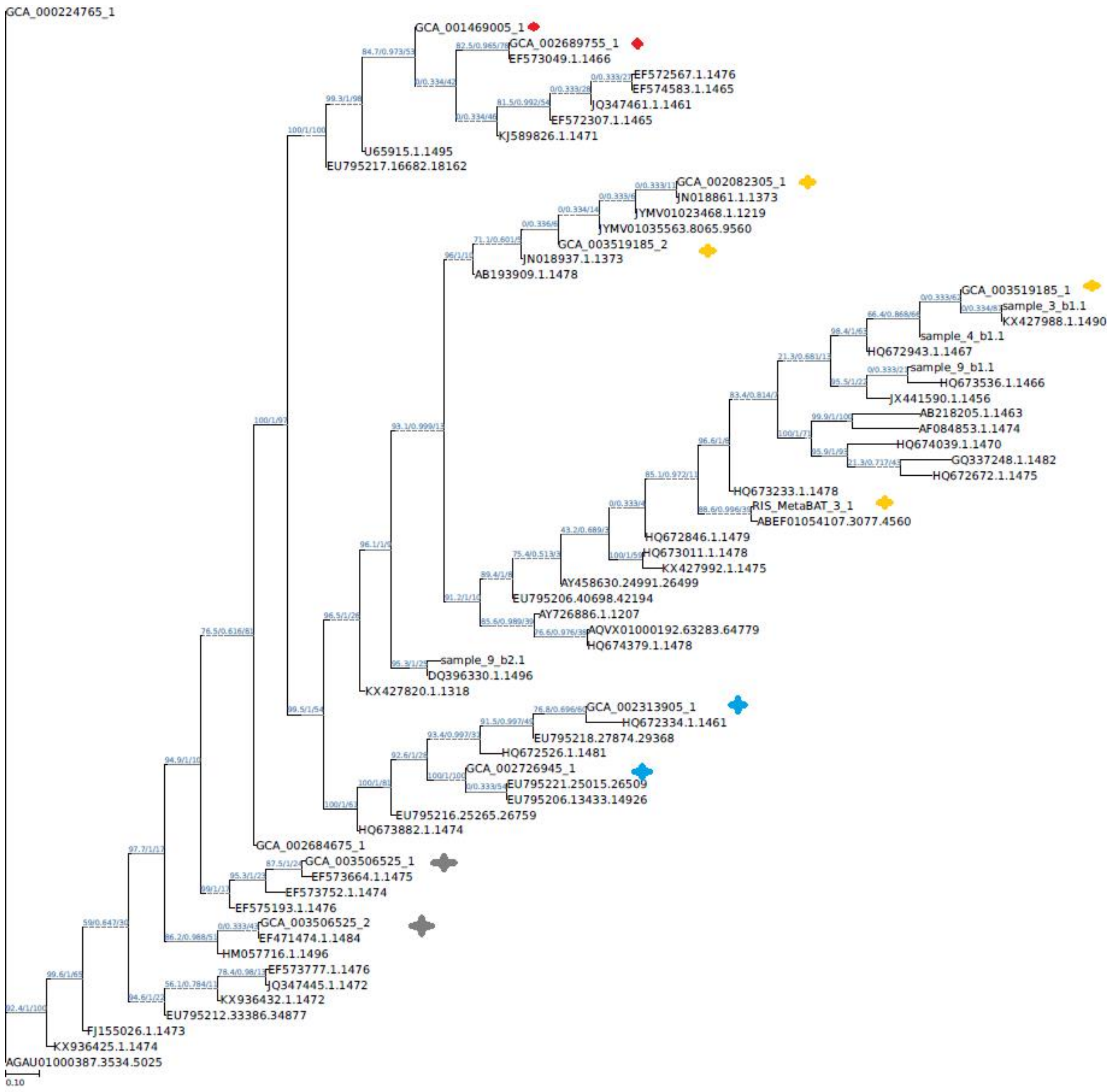

Figure S2. Maximum likelihood tree (IQtree) with 16s rRNA sequences. Red dots indicate sequences from MAGs belonging to Group 2C, blue dots to 2B, yellow dots to 2D and grey dots to 2A.

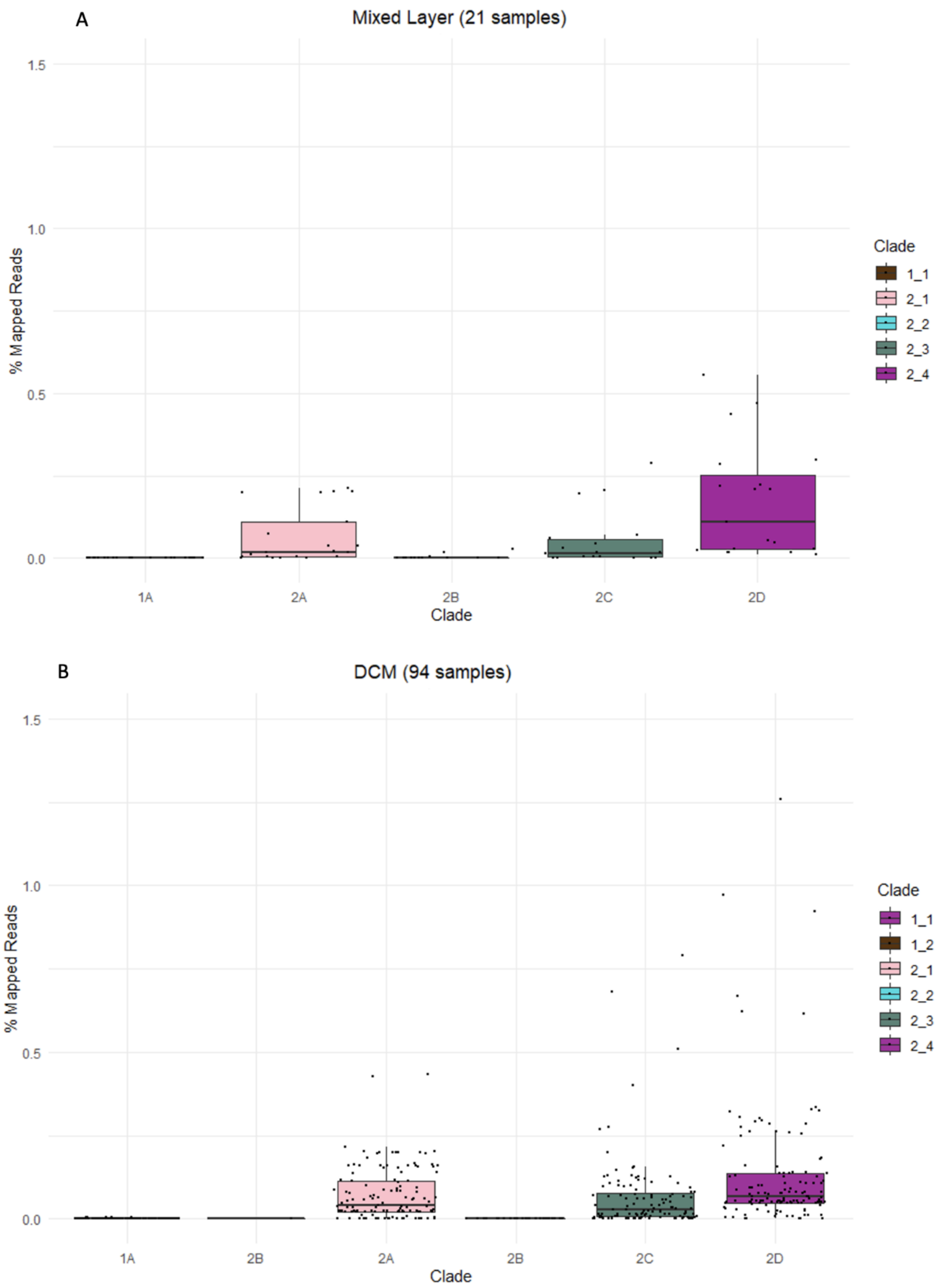

Figure S3. Bar plots showing the percentage of metatranscriptomic reads that mapped to the respective SAR324 clades relative to the total amount of reads present in each sample on the x-axis. 2A, 2C and especially 2D all appear to be active in the mixed layer (A) of the ocean as well as in the DCM (B).

Table S1. Origins and quality information of all the genomes used in this study.

ID	Environment	Completeness	Contamination	Genome size (mbp)	GC	score
GCA_000213335	South Atlantic sub-tropical gyre (station KN192-5-11)	50.69	0.0	2.16	41.1	Failed Threshold
GCA_000213355	North Pacific sub-tropical gyre (Hawaii Ocean Time-series (HOT) station ALOHA)	44.71	0.22	2.26	41.48	Failed Threshold
GCA_000224765	single cell collected from Scripps Research Pier at 6 m depth	70.44	0.16	4.26	43.32	71.94
GCA_000375785	-	25.35	3.97	1.39	39.28	Failed Threshold
GCA_000375805	-	25.63	0.0	0.86	40.69	Failed Threshold
GCA_001469005	Red Sea water column Station 34 - depth 10m	89.09	0.0	3.49	47.1	90.22
GCA_001627675	Red Sea water column Station 34 - depth 25m	72.17	2.94	2.54	47.46	59.14
GCA_001627685	Red Sea water column Station 22 - depth 50m	53.99	0.05	1.73	47.25	Failed Threshold
GCA_001627735	Red Sea water column Station 149 - depth 500m	86.3	0.0	3.12	42.82	88.68
GCA_001627755	Red Sea water column Station 34 - depth 258m	81.39	1.73	2.79	42.4	74.93
GCA_001627765	Red Sea water column Station 22 - depth 200m	53.97	4.09	2.12	43.25	Failed Threshold
GCA_001627795	Red Sea water column Station 22 - depth 500m	89.26	0.0	3.35	42.85	91.68
GCA_001627815	Red Sea water column Station 91 - depth 500m	86.15	0.05	3.05	42.85	88.18
GCA_001627835	Red Sea water column Station 169 - depth 500m	85.52	2.6	3.04	43.0	74.63
GCA_001627845	Red Sea water column Station 192 - depth 500m	89.54	0.0	3.17	42.89	91.93
GCA_001627865	Red Sea water column Station 108 - depth 500m	89.26	0.0	3.3	42.85	91.71
GCA_001627895	Red Sea water column Station 169 - depth 50m	50.79	0.84	1.37	46.86	Failed Threshold
GCA_001627915	Red Sea water column Station 22 - depth 25m	50.43	0.94	1.75	46.77	Failed Threshold
GCA_001627955	Red Sea water column Station 108 - depth 200m	66.99	0.91	2.26	42.52	64.79
GCA_001627975	Red Sea water column Station 34 - depth 200m	60.05	3.12	1.95	42.39	Failed Threshold
GCA_001781945	Rifle well CD01 at 16ft depth; 0.1 micron filter at time point D, USA: Rifle, CO	91.96	1.68	3.12	56.51	86.02
GCA_001781965	Rifle well CD01 at 16ft	48.02	0.0	1.97	48.3	Failed

	depth; 0.1 micron filter at time point D, USA: Rifle, CO					Threshold
GCA_001783655	Rifle well CD01 at 16ft depth; 0.1 micron filter at time point D, USA: Rifle, CO	91.96	1.68	3.09	56.5	86.02
GCA_001783695	Rifle well CD01 at 16ft depth; 0.1 micron filter at time point D, USA: Rifle, CO	91.83	1.68	3.14	49.59	84.97
GCA_001783715	Rifle well CD01 at 16ft depth; 0.1 micron filter at time point D, USA: Rifle, CO	91.96	1.68	3.08	56.53	86.01
GCA_002082285	hydrothermal plumes on the South Mid-Atlantic Ridge	31.82	0.0	0.98	41.22	Failed Threshold
GCA_002082295	hydrothermal plumes on the South Mid-Atlantic Ridge	89.51	0.0	2.93	42.41	91.71
GCA_002082305	hydrothermal plumes on the South Mid-Atlantic Ridge	90.35	0.0	2.8	42.3	92.57
GCA_002082535	hydrothermal plumes on the South Mid-Atlantic Ridge	61.89	0.05	1.44	42.94	Failed Threshold
GCA_002291585	marine	86.66	0.84	3.83	45.59	84.55
GCA_002313905	marine	81.27	0.84	3.15	55.43	79.13
GCA_002327995	marine	87.04	0.84	4.04	45.73	85.02
GCA_002328985	marine	86.78	1.73	4.07	45.72	80.31
GCA_002401295	Atlantic Ocean: North Pond, oxic subseafloor aquifer	69.22	2.95	3.08	41.71	55.67
GCA_002420365	marine	69.3	0.84	2.47	46.24	67.33
GCA_002450295	marine	68.29	0.11	3.12	45.93	69.86
GCA_002500045	marine	83.44	4.76	3.58	45.49	62.39
GCA_002683655	Chile-Peru Current Coastal Province, 5-1000m (TARA)	80.16	2.33	3.15	46.19	71.0
GCA_002683785	Chile-Peru Current Coastal Province, 5-1000m (TARA)	85.57	1.68	3.64	46.54	80.23
GCA_002684675	Chile-Peru Current Coastal Province, 5-1000m (TARA)	62.83	2.1	2.32	45.51	Failed Threshold
GCA_002685535	Chile-Peru Current Coastal Province, 5-1000m (TARA)	87.85	2.75	7.29	46.09	78.79
GCA_002685875	Northwest Arabian Sea Upwelling Province, 5-1000m (TARA)	66.06	0.84	1.66	57.69	63.91
GCA_002687455	Northwest Arabian Sea Upwelling Province, 5-1000m (TARA)	58.51	0.84	2.08	42.61	Failed Threshold
GCA_002689755	Mediterranean Sea, 5-1000m (TARA)	65.12	0.85	2.05	46.5	62.74
GCA_002690525	Mediterranean Sea, 5-1000m (TARA)	80.24	1.68	3.12	44.81	73.98

GCA_002699765	North Atlantic Ocean , 5-1000m (TARA)	62.02	0.93	2.08	56.89	Failed Threshold
GCA_002704555	Mediterranean Sea, 5-1000m (TARA)	69.42	1.05	1.97	38.89	66.38
GCA_002707655	South Atlantic Ocean , 5-1000m (TARA)	64.44	1.12	1.79	56.68	62.12
GCA_002716165	South Pacific Ocean , 5-1000m (TARA)	59.27	0.84	1.35	35.98	Failed Threshold
GCA_002717425	South Pacific Ocean , 5-1000m (TARA)	80.42	3.36	3.62	45.97	68.48
GCA_002725895	North Pacific Ocean , 5-1000m (TARA)	77.91	3.43	3.92	46.07	63.09
GCA_002726945	North Pacific Ocean , 5-1000m (TARA)	89.88	2.2	2.6	57.34	82.12
GCA_002730365	North Pacific Ocean , 5-1000m (TARA)	54.22	0.84	1.74	46.47	Failed Threshold
GCA_002753255	Australia: Punkally Creek Sediment	78.03	0.96	5.06	41.83	74.65
GCA_003506525	marine	72.8	0.95	3.48	44.67	70.33
GCA_003519185	Neamphius huxleyi metagenome	68.15	0.0	2.13	40.87	69.12
GCA_003542095	marine	60.07	0.0	1.95	42.23	Failed Threshold
GCA_003447105	marine	61.28	0.05	1.92	37.44	Failed Threshold
GCA_003508535	marine	51.11	0.0	2.24	55.22	Failed Threshold
GCA_003505015	hydrothermal vent	75.96	3.15	2.71	41.51	63.35
GCA_003507175	hydrothermal vent	60.27	0.0	2.31	36.46	Failed Threshold
GCA_003497305	hydrothermal vent	72.2	0.84	2.49	39.1	70.87
GCA_003541985	marine	67.36	0.84	2.48	44.62	64.09
GCA_003510845	marine	66.69	1.68	2.14	42.57	60.23
14_54	Red Sea brine pool	87.34	0.17	3.45	43.43	87.72
14_55	Red Sea brine pool	87.02	0.0	2.85	42.74	89.24
RIS_MetaBAT_11f-contigs	Ross Ice Shelf Antarctica	79.08	0.89	2.47	57.09	77.09
RIS_MetaBAT_3f-contigs	Ross Ice Shelf Antarctica	81.27	1.01	1.93	40.12	77.69
SAR324_MaxBin_bins.001	Malaspina Deep Sea Samples	91.15	2.69	2.61	42.46	80.38
SAR324_MaxBin_bins.003	Malaspina Deep Sea Samples	91.26	0.63	2.77	42.98	89.6
SAR324_MetaBAT_bins.001	Malaspina Deep Sea Samples	86.22	0.0	2.34	43.17	88.3
sample_7_b1	45° N, 178° E, Deep Sea	65.19	1.58	1.8	40.91	59.48

Table S2. Presence/Absence matrix from selected genes in all our MAGs. 0 means the gene is absent, 1 that it is present. This matrix is based on the myrast annotations. The columns represent the following genomes: X1=GCA_002401295; X2 = CA_001781945; X3 = GCA_001783695; X4 = GCA_002753255; X5 = GCA_002683655, X6 = GCA_003506525; X7=GCA_002690525; X8 = GCA_003541985; X9 = GCA_002726945; X10 = GCA_002313905; X11=RIS_MetaBAT_11; X12 = GCA_002327995; X13 = GCA_001469005; X14 =GCA_001627675; X15=GCA_002689755; X16 = GCA_001627845; X17 = GCA_002082305; X18 = GCA_003519185; X19=sample_7_b1, X20 = malaspina001; X21 = malaspina003, X22 = RIS_MetaBAT_3; X23=GCA_002704555, X24 = GCA_002685535; X25 =14_54

Gene abbreviations are explained at the end of the Table.

X	X1	X2	X3	X4	X5	X6	X7	X8	X9	X10	X11	X12	X13	X14	X15	X16	X17	X18	X19	X20	X21	X22	X23	X24	X25
AprA	0	0	0	0	0	0	0	0	0	0	0	1	0	0	0	1	1	1	0	0	0	0	0	0	1
AprB	0	0	0	0	0	0	0	0	0	0	0	1	0	0	0	1	1	1	0	0	0	0	0	0	1
Dsr_a	0	1	1	0	0	0	0	0	0	0	0	1	0	0	0	0	0	0	0	0	0	0	0	0	1
Dsr_b	0	1	1	0	0	0	0	0	0	0	0	1	0	0	0	0	0	0	0	0	0	0	0	0	1
sat	1	1	1	0	0	0	0	0	0	0	0	0	0	0	0	0	0	0	0	0	0	0	0	0	0
Fd_sulred	1	0	1	1	0	0	0	0	0	0	1	0	0	0	0	1	1	0	0	1	1	0	0	1	0
SoxY	0	0	0	0	1	1	0	0	0	1	0	1	1	0	0	1	1	0	1	0	1	1	1	1	1
SoxX	0	0	0	0	1	1	0	0	0	0	0	0	0	0	0	0	0	0	0	0	0	0	0	0	0
SoxA	0	0	0	0	1	1	0	0	0	0	0	0	0	0	0	0	0	0	0	0	0	0	0	0	1
SoxZ	0	0	0	0	1	1	0	0	0	0	0	0	1	0	0	0	0	0	0	0	0	0	0	0	1
SulP	0	0	1	1	0	0	0	0	0	0	0	1	0	0	0	0	0	0	0	0	0	0	0	0	0
Acsb_a_b	0	0	0	0	0	0	0	0	0	1	0	1	0	1	1	1	0	0	0	0	0	0	0	1	1
CoxL_med	0	0	0	0	1	1	1	1	1	1	1	1	1	1	1	1	1	1	0	0	1	0	1	0	1
CoxL_sml	0	0	0	0	1	1	1	1	1	1	1	1	1	1	1	1	1	1	1	0	1	0	1	0	1
CoxL_lrg	0	0	0	0	1	1	1	0	1	1	1	1	1	1	1	1	1	1	1	1	1	1	1	0	1
CoxL_D	0	1	0	0	1	1	1	1	1	1	1	1	1	1	0	1	1	0	1	0	1	1	0	0	1
CoxL_E	0	0	0	0	1	1	1	1	1	1	0	1	1	1	0	1	1	1	0	1	1	0	1	1	0
CoxL_I	0	0	0	0	1	1	0	0	0	1	1	1	1	1	1	1	1	1	1	0	0	0	0	0	0
ForDH	0	0	0	0	0	0	0	0	1	1	1	1	1	1	1	1	1	1	1	1	1	1	1	1	1
FormLig	0	0	0	0	0	0	0	0	1	1	1	0	1	1	1	1	1	1	1	1	1	1	0	1	1
MetTHF	0	1	1	1	0	1	1	0	1	1	0	1	1	0	1	1	1	1	1	1	1	1	1	1	1
MetRdc	0	1	1	1	1	1	1	0	1	1	1	1	1	1	1	1	1	1	1	1	1	1	1	1	1
acsE	1	1	1	1	1	1	1	0	1	1	1	1	1	1	0	1	1	1	1	1	1	1	1	1	1
aCoA	1	1	1	1	1	1	1	1	0	1	1	1	1	1	1	1	1	1	1	1	1	1	1	1	1
Hyd_lrg	0	1	1	1	0	0	1	0	0	0	0	1	0	0	0	0	0	0	0	0	0	0	0	0	0
Hyd_sml	0	1	1	0	0	0	1	0	0	0	0	1	0	0	0	0	0	0	0	0	0	0	0	0	0
CoBCoM_b	0	0	0	0	0	0	0	0	0	0	0	1	0	0	1	1	0	0	0	0	0	0	0	0	0
CoBCoM_a	0	0	0	0	0	0	0	0	0	0	0	1	0	0	1	1	1	1	0	0	0	0	0	0	1
CoBCoM_c	0	0	0	0	0	0	0	0	0	0	0	1	0	0	1	1	0	0	0	0	0	0	0	0	0
NirK	0	0	0	0	0	0	0	0	0	0	0	0	0	0	0	0	0	0	0	0	0	0	0	0	0
Nir	0	0	0	0	0	0	0	0	0	0	0	0	0	0	0	0	0	0	0	0	0	0	0	0	0
NirS_sml	0	0	0	0	0	0	0	0	0	0	0	0	0	0	0	0	0	0	0	0	0	0	0	0	0
NirS	1	0	0	0	0	0	0	0	0	0	0	0	0	0	0	0	0	0	0	0	0	0	0	0	1
NitNADH	1	0	0	0	0	0	0	0	0	0	0	0	0	0	0	0	0	0	0	0	0	0	0	0	0
NitTrans	1	0	0	0	0	0	0	0	1	0	0	1	0	0	0	1	0	0	1	1	1	0	0	1	0
DmdA	0	0	0	0	0	0	0	0	0	0	0	1	1	1	0	0	0	0	0	0	0	0	1	0	0
Rbsco_lrg	0	0	0	0	0	0	0	0	0	0	0	0	0	0	0	1	0	1	0	1	1	0	0	1	1
Rbsc_sml	0	0	0	0	0	0	0	0	0	0	0	0	0	0	0	1	0	1	0	1	0	0	0	1	1
CbbQ	0	0	0	0	0	0	0	0	0	0	0	0	0	0	0	1	0	1	0	1	0	0	0	1	1
CbbO	0	0	0	0	0	0	0	0	0	0	0	0	0	0	0	1	0	1	0	1	1	1	0	1	1
Trnskt	0	1	1	1	0	1	1	1	0	1	1	1	1	1	1	1	1	1	0	1	0	1	0	1	1

AldoI	0	0	0	0	1	1	1	1	0	0	0	1	1	1	1	1	1	1	1	0	0	0	0	0	0
AldoII	0	1	1	0	1	0	0	0	1	1	1	0	0	0	0	1	1	1	0	1	0	1	0	1	1
Cal1	0	0	0	0	0	0	0	0	0	0	0	0	0	0	0	1	0	0	0	1	0	1	0	1	1
Cal2	0	1	1	1	1	1	1	1	1	1	1	1	1	1	0	1	1	1	0	1	1	1	1	1	1
RibEmi	1	1	1	1	1	1	1	0	1	1	1	1	1	1	1	1	1	0	0	1	1	1	0	1	1
Cal3A	0	0	0	1	0	1	1	1	0	1	1	1	1	1	1	1	1	1	1	1	1	1	1	1	1
Cal3B	1	1	1	0	0	0	0	0	0	0	0	0	0	0	0	0	0	0	0	0	0	0	0	0	0
PhyCoA	1	0	0	0	1	1	1	1	1	1	1	1	1	1	1	1	1	1	1	1	1	1	1	1	1
Prot	0	0	0	0	1	1	1	1	0	0	0	0	0	0	0	0	0	0	0	0	0	0	0	0	0
BenzLig	0	0	0	0	0	0	0	0	0	0	0	1	1	1	1	1	1	1	1	0	0	1	1	1	0
BoxB	0	0	0	0	0	0	0	0	0	1	1	1	1	1	1	1	1	1	1	1	1	1	1	1	0
BoxC	0	0	0	0	0	0	0	0	0	0	1	1	1	1	1	1	1	1	0	1	1	1	1	1	0
BoxE	1	0	0	0	0	0	0	0	0	1	0	0	1	1	0	1	1	1	0	1	1	1	1	0	0
BrngHyd	0	0	0	0	0	0	0	0	0	0	0	0	0	0	0	1	1	1	0	1	1	1	1	1	0
pcaF	0	0	0	0	0	0	0	0	0	1	0	1	1	0	0	0	1	1	0	1	1	1	1	0	0
iorA	0	0	0	0	1	1	1	1	1	0	1	1	1	1	0	1	1	1	1	1	0	1	1	0	1
iorB	0	0	0	0	1	1	1	1	1	0	1	1	1	1	0	1	1	1	1	1	0	1	1	0	1
PaaI	1	0	0	0	0	0	0	0	0	1	1	0	0	0	0	0	1	1	0	0	0	0	0	0	1
PaaD	0	0	0	0	0	0	0	0	0	1	1	1	1	0	0	1	0	0	0	0	0	0	0	1	0
NADHUbi	0	0	0	1	1	1	1	1	1	1	1	1	1	1	0	1	1	1	0	0	1	1	0	1	1
SuccDeh	1	1	1	1	1	1	1	1	1	1	1	1	1	1	1	1	1	1	1	1	1	1	1	1	1
c553	0	0	0	1	1	1	0	0	1	1	1	1	1	1	1	1	1	1	1	1	1	1	0	1	1
c4	0	0	0	0	1	0	1	1	1	1	1	1	1	1	1	1	1	1	1	1	1	1	1	1	1
c2	1	0	0	1	0	0	0	0	0	0	0	0	0	0	0	0	0	0	0	0	0	0	0	0	1
c551	1	1	1	0	0	0	0	0	0	0	0	1	1	1	0	0	0	0	0	0	0	0	0	0	1
b558	0	0	0	1	1	1	1	0	1	1	0	1	1	1	1	1	1	1	1	1	1	1	1	1	1
fcC	0	0	0	0	0	1	0	0	0	1	0	0	0	0	0	1	0	1	0	1	0	0	0	0	1
cbb3	0	0	0	0	0	0	0	0	0	0	0	0	0	0	0	0	0	0	0	0	0	0	0	0	0
cheA	1	1	1	1	1	1	1	1	0	0	0	1	1	1	1	1	0	0	1	1	1	0	1	1	1
cheW	1	1	1	1	1	1	1	1	0	0	0	1	1	1	1	1	0	0	0	0	1	0	1	0	1
cheB	1	1	1	1	1	1	1	1	1	0	0	1	1	1	1	1	0	0	0	0	1	0	1	1	1
cheY	1	1	1	1	1	1	1	1	1	0	0	0	0	0	0	1	1	1	1	1	1	0	0	0	1
fliM	1	1	1	1	1	1	1	1	1	1	1	1	1	1	1	1	1	1	1	1	1	1	1	1	1
CtxP1	1	1	1	1	1	1	1	1	0	1	0	1	1	1	1	1	0	0	1	1	1	1	1	1	1
AceKin	0	1	1	1	0	0	0	0	0	0	0	1	1	1	0	0	0	0	0	0	0	0	0	0	0
Met1	0	0	0	0	0	0	0	0	0	0	0	0	0	0	0	0	0	0	0	0	0	0	0	1	0
Met2	0	0	0	0	0	1	1	0	0	0	0	1	1	0	0	0	0	0	0	0	0	0	0	0	0
Met3	1	1	1	1	1	0	1	1	1	1	0	1	1	1	1	0	1	1	1	0	1	1	1	1	1
Met4	0	1	1	1	1	0	1	0	1	1	0	1	1	0	0	0	0	0	0	0	0	0	0	0	0
Met5	1	1	1	1	1	1	1	0	1	1	1	1	1	1	1	1	1	0	0	1	1	1	0	1	1
AlcDeg	0	0	0	0	0	1	0	0	0	0	0	1	1	1	0	1	1	0	1	0	1	1	0	1	0

ABCS	0	0	0	1	0	1	0	0	0	0	0	1	1	0	0	1	0	1	0	1	0	0	0	0	1
ABCOP	1	1	1	1	1	1	1	1	1	1	1	1	1	1	1	1	1	1	0	0	1	0	0	1	1
ABCAA	1	1	1	1	1	1	0	1	0	1	1	1	1	1	1	1	1	1	1	1	1	1	1	0	1
ABCLPS	0	1	1	1	1	0	1	1	0	1	1	1	1	1	1	1	0	0	1	0	0	0	0	1	1
TCA1	0	1	1	1	0	0	0	0	0	0	0	0	1	1	0	0	1	1	1	1	1	1	1	1	1
TCA2	0	0	0	0	1	1	1	1	0	0	0	0	0	0	0	0	0	0	0	0	0	0	0	0	0
TCA3	0	0	0	0	0	0	0	0	1	0	1	1	1	1	1	1	1	1	1	0	0	1	0	1	0
Gly1	1	1	1	1	1	0	1	1	1	1	0	1	1	1	1	0	1	1	1	0	1	1	1	1	1
Gly2	1	1	0	1	1	1	1	0	0	0	0	1	1	1	1	1	1	1	1	1	1	1	1	1	1
Gly3	0	1	1	0	1	0	0	0	1	1	1	0	0	0	0	1	1	1	0	1	0	1	0	1	1
Gly4	0	0	0	0	1	1	1	1	0	0	0	1	1	1	1	1	1	1	1	0	0	0	0	0	0
Gly5	0	1	1	1	1	1	1	0	1	1	1	1	1	1	1	1	1	1	1	1	1	1	0	1	1
Gly6	1	0	0	0	0	0	0	0	1	1	0	1	0	0	0	1	0	0	1	1	0	0	1	1	0
FormaldUse	0	0	0	1	1	0	1	0	1	1	1	1	1	0	1	1	1	1	0	1	1	1	0	1	1
CytC	1	0	0	1	1	1	0	1	1	1	1	1	1	1	0	1	1	1	0	1	1	1	1	1	1

Gene abbreviations in Supplementary Table 2 stand for:
Apr_mmb = Adenylylsulfate reductase membrane anchor
Apr_put = Phosphoadenylyl-sulfate reductase thioredoxin / Adenylyl-sulfate reductase thioredoxin
AprA = Adenylylsulfate reductase alpha-subunit
AprB = Adenylylsulfate reductase beta-subunit
Dsr_a = Dissimilatory sulfite reductase, alpha subunit
Dsr_b = Dissimilatory sulfite reductase, beta subunit
sat = Sulfate adenylyltransferase subunit 1 / Adenylylsulfate kinase
Dsr_put = Probable sulfite reductase
Fd_sulred = Ferredoxin--sulfite reductase
SoxY = Sulfur oxidation protein SoxY
SoxX = Sulfur oxidation protein SoxX
SoxA = sulfur oxidation protein SoxA
SoxZ = Sulfur oxidation protein SoxZ
SulP = Sulfate permease
Acsb_a_b Acetyl-CoA synthetase (ADP-forming) alpha and beta chains, putative
Acsb_a = Acetyl-CoA synthetase (ADP-forming) alpha chain
CoxL_med = Carbon monoxide dehydrogenase medium chain
CoxL_sml = Carbon monoxide dehydrogenase small chain
CoxL_lrg = Carbon monoxide dehydrogenase large chain
CoxL_D = carbon monoxide dehydrogenase D protein
CoxL_E = carbon monoxide dehydrogenase E protein
CoxL_I = Carbon monoxide dehydrogenase form I, large chain
FormLig = Formate--tetrahydrofolate ligase
MetTHF = Methylenetetrahydrofolate dehydrogenase
MetRdc = 5,10-methylenetetrahydrofolate reductase
acsE = 5-methyltetrahydrofolate--homocysteine methyltransferase
ForDH = tungsten-containing formate dehydrogenase alpha subunit
aCoA = Acetyl-coenzyme A synthetase
Hyd_lrg = Uptake hydrogenase large subunit
Hyd_sml = Uptake hydrogenase small subunit precursor
CoBCoM_b = CoB--CoM heterodisulfide reductase subunit B

CoBCoM_a = CoB--CoM heterodisulfide reductase subunit A
CoBCoM_c = CoB--CoM heterodisulfide reductase subunit C
NirK = Copper-containing nitrite reductase
Nir = Ferredoxin--nitrite reductase
NirS_sml = Cytochrome c nitrite reductase, small subunit NrfH
NirS = Cytochrome cd1 nitrite reductase
NitNADH = Nitrite reductase [NAD
NitTrans = Nitrate/nitrite transporter
DmdA = DMSP demethylase
Rbsco_lrg = Ribulose bisphosphate carboxylase large chain
Rbsc = Ribulose bisphosphate carboxylase
Rbsc_sml = Ribulose bisphosphate carboxylase small chain
CbbQ = Rubisco activation protein CbbQ
CbbO = Rubisco activation protein CbbO
CbbR = Rubisco operon transcriptional regulator CbbR
Trnskt = Transketolase
AldoI = Fructose-bisphosphate aldolase class I
AldoII = Fructose-bisphosphate aldolase class II
Cal1 = Uridine kinase
Cal2 = Phosphoglycerate kinase
Cal3A = Ribose 5-phosphate isomerase A
Cal3B = Ribose 5-phosphate isomerase B
RibEmi = Ribulose-phosphate 3-epimerase
PhyCoA = Phytanoyl-CoA dioxygenase
Prot = Proteorhodopsin
BenzLig = Benzoate-CoA ligase
BoxB = Benzoyl-CoA oxygenase component B
BoxC = Benzoyl-CoA-dihydrodiol lyase
BoxE = Acetyl-CoA acetyltransferase
BrngHyd = benzoate degradation ring-cleavage hydrolase
pcaF = pcaF: 3-oxoadipyl-CoA thiolase
iorA = Isoquinoline 1-oxidoreductase alpha subunit
iorB = Isoquinoline 1-oxidoreductase beta subunit
PaaI = putative protein PaaI, possibly involved in aromatic compounds catabolism
PaaD = Phenylacetic acid degradation protein PaaD, thioesterase
NADHUbi = NADH ubiquinone oxidoreductase chain A
SuccDeh = Succinate dehydrogenase iron-sulfur protein
c553, "Putative diheme cytochrome c-553
c4 = Cytochrome c4
c2 = Probable cytochrome c2
c551 = Cytochrome c551 peroxidase
b558 = Succinate dehydrogenase cytochrome b558 subunit
fcC = Sulfide dehydrogenase [flavocytochrome C] flavoprotein chain precursor
cbb3 = probable cytochrome oxidase
cheA = Signal transduction histidine kinase CheA
cheW = Positive regulator of CheA protein activity
cheB = Chemotaxis response regulator protein-glutamate methylesterase CheB
cheY = Chemotaxis regulator - transmits chemoreceptor signals to flagelllar motor components CheY
fliM = Flagellar motor switch protein FliM
CtxP1 = Methyl-accepting chemotaxis protein I

AceKin = Acetate kinase
Met1 = 6-phosphogluconate dehydrogenase, decarboxylating
Met2 = 6-phosphogluconolactonase
Met3 = Glucose-6-phosphate isomerase
Met4 = Transaldolase
Met5 = Ribulose-phosphate 3-epimerase
AlcDeg = 3-hydroxyisobutyrate dehydrogenase
ABCS = ABC-type sugar transport system, permease component
ABCOP = Oligopeptide ABC transporter, periplasmic oligopeptide-binding protein OppA
ABCAA = Branched-chain amino acid ABC transporter, amino acid-binding protein
ABCLPS = Lipopolysaccharide ABC transporter, ATP-binding protein LptB
TCA1 = Isocitrate dehydrogenase [NADP]
TCA2 = Ketoglutarate semialdehyde dehydrogenase
TCA3 = Propionyl-CoA carboxylase carboxyl transferase subunit
Gly1 = Glucose-6-phosphate isomerase
Gly2 = 6-phosphofructokinase
Gly3 = Fructose-bisphosphate aldolase class II
Gly4 = Fructose-bisphosphate aldolase class I
Gly5 = Triosephosphate isomerase
Gly6 = ROK family Glucokinase with ambiguous substrate specificity
FormaldUse = Serine hydroxymethyltransferase
CytC = Cytochrome c oxidase polypeptide I
